# Transcriptome and QTL mapping analyses of major QTL genes controlling glucosinolate contents in vegetable- and oilseed-type *Brassica rapa* plants

**DOI:** 10.3389/fpls.2022.1067508

**Published:** 2023-01-18

**Authors:** Jin A. Kim, Heewon Moon, Hyang Suk Kim, Dasom Choi, Nan-Sun Kim, Juna Jang, Sang Woo Lee, Adji Baskoro Dwi Nugroho, Dong-Hwan Kim

**Affiliations:** ^1^Department of Agricultural Biotechnology, National Institute of Agricultural Science, Rural Development Administration, Jeonju, Jeollabuk-do, Republic of Korea; ^2^Department of Plant Science and Technology, Chung-Ang University, Anseong, Republic of Korea

**Keywords:** glucosinolate, *Brassica rapa*, QTL mapping, *BrMYB28.1*, transcriptome

## Abstract

Glucosinolates (GSLs) are secondary metabolites providing defense against pathogens and herbivores in plants, and anti-carcinogenic activity against human cancer cells. Profiles of GSLs vary greatly among members of genus *Brassica*. In this study, we found that a reference line of Chinese cabbage (*B. rapa* ssp. *pekinensis*), ‘Chiifu’ contains significantly lower amounts of total GSLs than the oilseed-type *B. rapa* (*B. rapa* ssp. *trilocularis*) line ‘LP08’. This study aimed to identify the key regulators of the high accumulation of GSLs in *Brassica rapa* plants using transcriptomic and linkage mapping approaches. Comparative transcriptome analysis showed that, in total, 8,276 and 9,878 genes were differentially expressed between ‘Chiifu’ and ‘LP08’ under light and dark conditions, respectively. Among 162 *B. rapa* GSL pathway genes, 79 were related to GSL metabolism under light conditions. We also performed QTL analysis using a single nucleotide polymorphism-based linkage map constructed using 151 F_5_ individuals derived from a cross between the ‘Chiifu’ and ‘LP08’ inbred lines. Two major QTL peaks were successfully identified on chromosome 3 using high-performance liquid chromatography to obtain GSL profiles from 97 F_5_ recombinant inbred lines. The MYB-domain transcription factor gene *BrMYB28.1* (Bra012961) was found in the highest QTL peak region. The second highest peak was located near the 2-oxoacid-dependent dioxygenase gene *BrGSL-OH.1* (Bra022920). This study identified major genes responsible for differing profiles of GSLs between ‘Chiifu’ and ‘LP08’. Thus, our study provides molecular insights into differences in GSL profiles between vegetative- and oilseed-type *B. rapa* plants.

## 1 Introduction

Secondary metabolites of plants have diverse functions throughout the plant’s lifespan. A lack of plant secondary metabolites does not lead to immediate death, but can affect the survival and reproduction of plant species over the long term (Isah, 2019). Many secondary metabolites play important roles in plant defense systems against a variety of environmental stresses including salt, drought, heat, wounding, and attacks from pathogens (Dixon, 2001). Furthermore, plant secondary metabolites determine important aspects of human food quality, such as taste and flavor (Verpoorte and Memelink, 2002).

Glucosinolates (GSLs), a type of plant secondary metabolite, are mainly produced in crop plants of the Brassicaceae family, and help the plants resist stresses including attack by insects and herbivores (Halkier and Gershenzon, 2006; Hopkins et al., 2009). More than 130 GSLs have been identified in the Brassicaceae family (Agerbirk and Olsen, 2012; Thinh Nguyen et al., 2020). GSLs are not only important molecules for plant defense, but are also reported to have anti-cancer, anti-inflammatory, and other health benefits in humans (Verhoeven et al., 1996; Keck and Finley, 2004). Some GSLs, such as glucoraphanin (GRA), glucoalyssin (GAS), gluconapin (GNP), neoglucobrassicin (NGB), and gluconasturtiin (GNT), are beneficial, whereas hydrolysis products from progoitrin (PRO), epiprogoitrin (epiPRO), and gluconapoleiferin (GNL) can cause goiter in animals (Sønderby et al., 2010b). GSLs are derived from amino acids and can be divided into aliphatic (derived from Met, Leu, Ala, Ile, and Val), indolic (derived from Trp), and benzenic (derived from Phe and Tyr) GSLs based on their amino acid precursors (Grubb and Abel, 2006).

Structural differences among GSL compounds are driven mainly by variations of the genes involved in the initial side chain elongation and secondary modification stages (Halkier and Gershenzon, 2006). GSL biosynthetic processes have been intensively studied in the model plant *Arabidopsis thaliana* (Sønderby et al., 2010a). A small subgroup of R2R3-type myeloblastosis (MYB) transcription factors (TFs) plays an important role in the regulation of GSL metabolism. For example, *Arabidopsis* has three *MYB* TF genes (*MYB28*, *MYB29*, and *MYB76*) controlling aliphatic GSL biosynthesis, and another three (*MYB34*, *MYB51*, and *MYB122*) regulating indolic GSL biosynthesis (Gigolashvili et al., 2007; Hirai et al., 2007; Sønderby et al., 2007). Double mutants of the *MYB28* and *MYB29* genes, designated *myb28;myb29*, exhibited severe reduction of aliphatic GSLs (Li et al., 2013). Another TF, the DNA-binding with one finger (DOF) domain-containing TF *OBP2* (also referred to as *AtDof1.1*, AT1G07640), positively regulated aliphatic GSL biosynthesis (Skirycz et al., 2006). All of these TFs function as positive regulators of GSL biosynthesis.

In addition to these TFs, many genes encoding catalytic enzymes are involved in the aliphatic and indolic GSL biosynthetic pathways. GSL biosynthesis generally consists of four stages: the ‘chain elongation’ stage of amino acid precursors such as methionine (Met) and phenylalanine (Phe); the ‘core structure formation’ stage; the ‘secondary modification’ stage; and the ‘breakdown’ stage (Wittstock and Halkier, 2002). Initial stage ‘side-chain elongation’ starts from the side chain elongation of precursor amino acids, which involves the enzymes METHYLTHIOALKYL MALATE SYNTHASE 1 (MAM1), MAM2, MAM3, BRANCED-CHAIN AMINOTRANSFERASE 3 (BCAT3), BRANCED-CHAIN AMINOTRANSFERASE 4 (BCAT4), BILE ACID TRANSPORTER 5 (BAT5), IPMDH1 (ISOPROPYLMALATE DEHYDROGENASE 1), IPMI1 (ISOPROPYLMALATE ISOMERASE 1) and IPMI2. GSLs with various types of side-chain can be produced depending on allelic variations and/or even different developmental tissues. After the ‘side chain elongation’ stage, CYTOCHROME P450 enzymes, including CYP79F1 and CYP79F2 (aliphatic GSL pathway) and CYP79B2 and CYP79B3 (indolic GSL pathway) convert elongated amino acids into aldoxime which is further converted into aci-nitro compounds by CYP83A1 (aliphatic GSLs) and CYP83B1 (indolic GSLs). Subsequently, the aci-nitro compound is further modified through a series of catalytic activities by the enzymes SUR1, UGT74B1, UGT74C1, and ST5a/b/c (SOT16/18/17). After ‘core structure formation’, desulfo-GSLs undergo the ‘secondary modification’ process which involves FLAVIN-MONOOXYGENASE GLUCOSINOLATE S-OXYGENASES (FMO GS-OXs), ALKENYL HYDROXALKYL PRODUCING 2 (AOP2), AOP3, and GLUCOSINOLATE HYDROXYLASE (GSL-OH) for aliphatic GSLs (Zhang et al., 2015; Kakizaki et al., 2017). The ‘secondary modification’ of indolic GSLs is processed by a small group of cytochrome P40 monooxygenase family proteins, including CYTOCHROME P450 MONOOXYGENASE 81 SUBUNIT 1 (CYP81F1) to CYP81F4, and INDOLE GLUCOSINOLATE O-METHYLTRANSFERASE 1 (IGMT1) and IGMT2. Later, GSL compounds are further hydrolyzed by plant enzymes, commonly called myrosinases including THIOGLUCOSIDE GLUCOHYDROLASE 1 (TGG1) to TGG5, EPITHIOSPECIFIER (ESP), and NITRILE SPECIFIER PROTEIN (NSP1) to NSP5 (aliphatic GSLs) and PENETRATION 2 (PEN2), PEN3, and CADMIUM SENSITIVE 1 (CAD1) (indolic GSLs).

It was previously reported that level of GSL compounds and the expression of GSL biosynthetic genes are positively affected by light (Schuster et al., 2006; Huseby et al., 2013). For instance, GSLs are highly synthesized during day time and significantly reduced in night time in a daily basis. Expression of MYB TF genes and their downstream GSL biosynthetic genes were also influenced by the presence of light. For example, expression of *MYB* TF genes (*MYB28*, *MYB29*, and *MYB76*) and GSL biosynthetic genes (*CYP79F1*, *SOT17*, *SOT18* etc.) involved in the aliphatic GSL biosynthesis were significantly reduced in the absence of light (Huseby et al., 2013). It indicated that light signaling positively stimulate expression of GSL pathway genes. Furthermore, light-stimulated increase of GSLs is also in an agreement with the fact that glucose and sulfur, two essential precursors for GSL biosynthesis are highly synthesized during day time by photosynthesis and sulfate assimilation, respectively (Koprivova and Kopriva, 2014).

During last decades, many studies investigated the GSL profiles of various *Brassica* crops, such as *Brassica rapa*, Chinese kale, broccoli, cabbage, and cauliflower (Vallejo et al., 2002; Xu et al., 2006; Bellostas et al., 2007; Padilla et al., 2007; Jia et al., 2009; Sun et al., 2011; Wang et al., 2012). For example, total 16 GSLs were identified in *B. rapa* from seeds and leaves of adult stage plant, which displayed two aliphatic GSLs, gluconapin (GNP) and glucobrassicanapin (GBN) most abundant. Indolic and aromatic GSLs levels were low and showed few differences among different *B. rapa* varieties (Padilla et al., 2007). Bellostas et al. (2007) have investigated the GSLs profiles of five varieties of *B. oleracea* (white cabbage, red cabbage, Savoy cabbage, broccoli, and cauliflower) in sprouting stage. The concentration of alkyl-GSLs in these *B. oleracea* cultivars decreased, whereas glucobrassicin (GBS) significantly increased throughout the sprouting period. In broccoli, GSLs were evaluated in several cultivars (Vallejo et al., 2002). Dominant GSLs in all broccoli cultivars were glucoraphanin (GRA) and glucobrassicin (GBS) (Vallejo et al., 2003; Schonhof et al., 2004; Wang et al., 2012). GSLs profiles of Chinese Kale (*Brassica oleracea* var. *acephala*) is only limitedly informed. Sun et al. (2011) investigated the GSLs in three edible parts of Chinese kale (sprout, tender rosette leaf, and bolting stem). Thirteen GSLs, including eight aliphatic GSLs, four indole GSLs, and one aromatic GSL were identified in Chinese kale. The aliphatic GSLs were the most abundant GSLs in Chinese kale, with gluconapin (GNP) being the most abundant individual GSLs.

*Brassica rapa* has many subspecies with marked morphological variations (Seo et al., 2017), including oilseed crop yellow sarson (*B.rapa* ssp. *trilocularis*) for the production of seed oil and leafy vegetable type Chinese cabbage (*B.rapa* ssp. *pekinensis*) for leaf consumption. It has great differences between both types in terms of phenotypic traits. One of the Chinese cabbage vegetable type, ‘Chiifu’ line is self-incompatible and flowers late, requiring vernalization for bolting, and has a wide leaf shape. Meanwhile, the yellow sarson, ‘LP08’ line is self-compatible and flowers rapidly, and has narrow leaves with serrated margins.

In *B. rapa*, recent study quantified GSL levels of the eight subspecies of *B. rapa* which exhibited variable GSL levels ranging from 4.42 μmol·g^−1^ dw of pak choi (*B. rapa* ssp. c*hinensis*) to 53.51 μmol·g^−1^ dw of yellow sarson (*B. rapa* ssp. *trilocularis*) (Seo et al., 2017). GSL profiling using the doubled haploid (DH) lines produced between high GSLs [*B. rapa* ssp *trilocularis* (yellow sarson)] and low GSLs [*B. rapa* ssp. c*hinensis* (pak choi)] parents identified GSL-specific recombinant block on A03 (12.9 Mb ~ 23.2 Mb) chromosome based on SNP and InDels (Soundararajan et al., 2021). Similar to previous reports (Seo et al., 2017; Soundararajan et al., 2021), we also found that total amounts of GSLs were significantly higher in the oilseed-type yellow sarson ‘LP08’ (*B. rapa* ssp. *trilocularis*) line than the vegetable-type Chinese cabbage ‘Chiifu’ (*B. rapa* ssp. *pekinensis*) line. In this study, to elucidate the major gene or genes responsible for the difference in GSL contents between these two lines, we employed two comprehensive approaches: comparative transcriptome and quantitative trait locus (QTL) mapping. This study reveals the molecular mechanisms underlying the differing GSL contents between these two inbred lines, i.e., the molecular mechanisms of GSL pathways in *B. rapa* plants.

## 2 Materials and methods

### 2.1 Plant materials and growth conditions

For transcriptome analysis, the Chinese cabbage (B. rapa ssp. pekinensis) ‘Chiifu’ inbred line and yellow sarson (B. rapa ssp. trilocularis) ‘LP08’ line were used in this study. For the mapping population, 151 individuals in the F5 generation of a *Brassica rapa* segregating population were obtained by crossing the ‘Chiifu’ and ‘LP08’ lines in the greenhouse of Rural Development Administration of Korea as described previously (Kim et al., 2017; Kim et al., 2022). This population was used for QTL mapping of GSL contents.

Considering that GSL content is highly affected by a diversity of environmental stresses, in this study, we wanted to exclude environmental stress factors as much as possible and identify genetic factor(s) contributing to different GSL amounts between Chiifu and LP08. Thus, we grew seedling plants in a plant culture dish containing MS media in an environment-controlled growth chamber (22°C, 16h light/8h dark photoperiod). For quantification of GSL contents, 97 F_5_ seedlings of recombinant inbred lines (RILs), as well as the parental lines ‘Chiifu’ and ‘LP08’, were used for extraction of GSLs. Seeds were surface-sterilized, spread on half-strength Murashige and Skoog agar medium and stored in the dark at 4°C for 3 days for stratification. Seedlings were grown for 1 week in a growth chamber at 22°C under long-day (LD) (16-h light/8-h dark) conditions. At least 5 seedling plants per line were harvested for high-performance liquid chromatography (HPLC) analysis.

### 2.2 Construction of genotyping-by-sequencing (GBS) libraries

All 151 F_5_ lines, as well as the two parental lines, were subjected to GBS using the Illumina NextSeq500 sequencing platform, as described previously (Kim et al., 2022). GBS libraries were sequenced on the NextSeq500 system (Illumina, USA) and single-end reads 150 bp in length were obtained. After sequencing of GBS libraries, the raw reads were de-multiplexed to sort samples using the GBSX tool (Herten et al., 2015). The *Brassica rapa* reference genome (Brassica_rapa.Brapa_1.0.dna.toplevel.fa) was obtained from the EnsemblPlants genome database (https://plants.ensembl.org/index.html). After de-multiplexing, single-end reads were mapped to the *B. rapa* reference genome using Bowtie2 (Langmead and Salzberg, 2012). For calling of single nucleotide polymorphism (SNP) variants, the Genome Analysis Toolkit (GATK) and Picard tools (McKenna et al., 2010) packages were used. Local realignment of reads was performed to correct misalignment resulting from the presence of indels, using the GATK ‘RealignerTargetCreator’ and ‘Indoleamine’ sequence data processing tools. Lastly, the GATK ‘HaplotypeCaller’ and ‘SelectVariants’ tools were used for SNP variant calling.

### 2.3 Linkage map construction and QTL mapping

The variant call format SNP data were transformed into the input format using customized code for the R/Qtl package (eGenome, Republic of Korea). Markers with a duplicated pattern or distorted segregation ratio, as estimated using the Chi-square test with Bonferroni correction, were removed. The est.map function of R/Qtl was used to construct the linkage map, with the Kosambi map function converting genetic distances into recombination fractions (*p*-value threshold of 1e-06, and EM iterations in 1000 times). Subsequently, the composite interval mapping function of the R/Qtl package was used for QTL mapping with the Kosambi function. Regions inferred from peak positions with local maximum limit of detection (LOD) values exceeding the threshold determined using 1,000 permutation tests were considered significant QTLs. To detect QTLs responsible for the differing GSL profiles of the two parental lines, the GSL contents of 97 F_5_ lines and the parental lines were measured and applied to the SNP linkage map. The LOD score threshold value for significance (*ɑ* = 0.05) was estimated based on 1,000 permutation tests. Peaks exceeding the estimated LOD threshold value were selected for further analysis.

### 2.4 Extraction of GSLs and HPLC

Plants were grown at 22°C under a 16-h light/8-h dark photoperiod. One-week-old plants were harvested at ZT4 (4 h after light on) and then immediately ground in liquid nitrogen. GSLs were extracted as desulfo-glucosinolates (DS-GSLs), as reported previously (Han et al., 2019). Approximately 500 mg of fresh sample was incubated with 70% methanol at 70°C for 25 min to deactivate myrosinases. Contents of DS-GSLs were analyzed through ultra-high-performance liquid chromatography (UHPLC; 3000 U-HPLC system; ThermoFisher Scientific, USA). As a standard, sinigrin (0.5mg/ml) injection was used in all analyses (Sigma-Aldrich, USA). The DS-GSLs were resolved with a C18 reverse phase column (Zorbax XDB-C18, 4.6 × 250 mm, 5-µm particle size; Agilent, USA) with a water and acetonitrile gradient system. Samples were injected and maintained at a flow rate of 0.5 ml/min. Peaks were identified using standard compounds (Phytoplan, Germany). The samples were analyzed independently (three replicates) and the results are presented in nmol/g based on fresh weight (FW).

### 2.5 Analysis of GSLs through liquid chromatography coupled to diode array detection and electrospray ionization mass spectrometry

DS-GSLs were analyzed using the Accela UHPLC system (ThermoFisher Scientific) fitted with an ion trap mass spectrometer (LTQ Velos Pro; ThermoFisher Scientific). The samples were resolved using a C18 reverse phase column (Zorbax XDB-C18, 4.6 × 250 mm, 5-μm particle size; Agilent) with water and acetonitrile as the mobile phase, and measured in negative ion mode ([M-H]-). Mass spectrometry was conducted with the following settings: capillary temperature, 275°C; capillary voltage, 5kV; source heater temperature, 250°C; sheath gas flow, 35 arb; auxiliary gas flow, 5 arb; and spectral scanning range, *m/z* 100–1,500.

### 2.6 RNA sequencing and library construction

Total RNA was extracted from 1-week-old seedlings grown at 22°C under light (16-h light/8-h dark photoperiod) or dark conditions. Three biological replicates were harvested at each time point and frozen in liquid nitrogen. Total RNA was extracted using the RNeasy Plant Mini Kit (QIAGEN, Germany) and subsequently treated with DNase I (NEB, USA) to remove contaminating genomic DNA. Purified total RNA was used for construction of RNA-seq libraries using the TruSeq RNA Sample Preparation Kit (Illumina) according to the manufacturer’s instructions. Paired-end sequencing was performed on the HiSeq 2500 system (Illumina).

### 2.7 Sequence alignment and analysis

Prior to alignment of RNA-seq reads to the *Brassica rapa* reference genome, FastQC software (http://www.bioinformatics.babraham.ac.uk/projects/fastqc) was employed to evaluate the quality of the RNA-seq reads. Reads with > 90% Q values > 30 were filtered and used only for genome alignment. The *B. rapa* FASTA genome file (Brassica_rapa.Brapa_1.0.dna.toplevel.fa) and gff3 file (Brassica_rapa.Brapa_1.0.54.gff3) were downloaded from the EnsemblPlants genome database. TopHat2 mapping software was employed with the default parameters for alignment of reads to the reference genome (Kim et al., 2013). Digital read counts were obtained using featureCounts (Liao et al., 2014) and subsequently analyzed for differentially expressed genes (DEGs) using edgeR. The cut-off for DEGs was a two-fold difference (p < 0.05). A multi-dimensional scaling (MDS) plot, correlation map, and PlotSmear results were produced using R software (ver. 3.6.1; https://www.rstudio.com/products/rpackages/) packages. The web-based tool VENNY was used to generate a Venn diagram (http://bioinformatics.psb.ugent.be/webtools/Venn/). The web-based tool ShinyGO (ver. 0.61) was used for Gene Ontology (GO) enrichment analysis (http://bioinformatics.sdstate.edu/go/). Hierarchical clustering heatmap analysis was performed using Cluster 3.0 (http://bonsai.hgc.jp/~mdehoon/software/cluster/software.htm) and the JAVA TreeView program (Saldanha, 2004). Mapping results were visualized with the Integrative Genomics Viewer (IGV) program of the Broad Institute (Thorvaldsdóttir et al., 2013).

### 2.8 RNA extraction and quantitative reverse transcription polymerase chain reaction analysis

Total RNA was extracted from 1-week-old seedlings grown at 22°C under light (16-h light/8-h dark photoperiod) or dark conditions using the RNeasy Plant Mini Kit (Qiagen). Extracted RNA was treated with DNase I (NEB) to remove contaminating genomic DNA. Approximately 5 µg of total RNA was used for cDNA synthesis with EasyScript reverse transcriptase (TransGen Biotech, China). qRT-PCR was performed using Solg 2× Real-Time PCR Smart Mix (SolGent, Republic of Korea) on a LineGene 9600 Plus Real-Time PCR system (BIOER, China), according to the manufacturer’s instructions. qRT-PCR was conducted under the following conditions: denaturation at 95°C for 12 min, followed by 50 cycles of amplification (95°C for 15 s, 60°C for 25 s, 72°C for 35 s) and sampling at 72°C. The relative transcript level of each gene was determined through comparison with that of *BrPP2Aa* (Bra012474), a housekeeping gene consistently expressed in our RNA-seq analysis. The primers were designed based on sequences obtained from the *B. rapa* genome database (BRAD; http://brassicadb.cn). The primers used for qPCR analysis are presented in **Supplementary Table S6**. Student’s *t*-test was used for statistical analysis (*P < 0.05, **P < 0.01, ***P < 0.001).

### 2.9 Sequencing of the *BrMYB28.1* and *BrGSL-OH.1* genes

PCR amplification was performed to clone two major QTL candidate genes, *BrMYB28.1* and *BrGSL-OH.1*, using gene-specific primers (**Supplementary Table S6**). PCR reaction was conducted with the following conditions: denaturation at 94°C for 5 min, followed by three initial cycles of amplification (94°C for 30 s, 55°C for 35 s, 72°C for 5 min), 33 additional cycles of amplification (94°C for 30 s, 64°C for 35 s, 72°C for 4 min 30 s) and a final extension step at 72°C for 10 min. The PCR product was extracted from the agarose gel after 1% agarose gel electrophoresis and then purified with the Dyne Power Gel Extraction Kit (Dynebio, Republic of Korea). The purified PCR products were inserted into the pPZP211 plant expression vector using the In-fusion HD cloning kit (Takara Bio, Japan). Entire genomic sequences of *BrMYB28.1* and *BrGSL-OH.1* were obtained through Sanger sequencing (Bionics, Republic of Korea) using the series of primers listed in **Supplementary Table S6**.

## 3 Results

### 3.1 Comparison of levels and compositions of GSLs between ‘Chiifu’ and ‘LP08’

**Figure 1:**
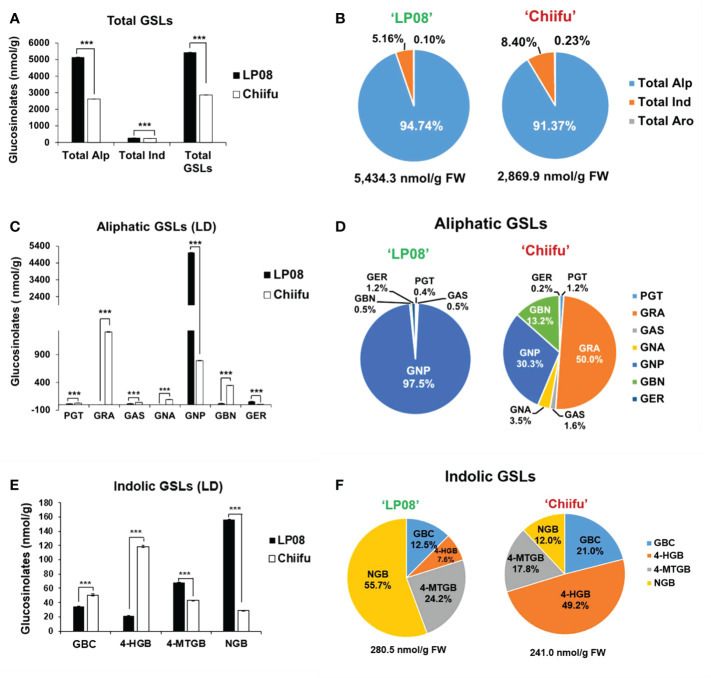
Measurement of GSL compounds in ‘Chiifu’ and ‘LP08’. **(A)** Comparison of total GSL amounts (aliphatic, indolic, and aromatic GSL compounds) in ‘Chiifu’ and ‘LP08’ plants grown under long-day **(LD)** growth conditions. **(B)** Pie charts showing aliphatic (Alp), indolic (Ind), and aromatic (Aro) GSL compounds as percentages of total GSL content. Aliphatic GSL compounds constituted the majority of the GSLs in both the ‘Chiifu’ and ‘LP08’ lines. **(C)** Bar graph showing the amounts of seven aliphatic GSL compounds in ‘Chiifu’ and ‘LP08’. Student’s *t*-test was used to calculate statistically significant differences (^***^p ≤ 0.001). **(D)** Pie charts showing the amounts of individual aliphatic GSLs in ‘Chiifu’ and ‘LP08’. PGT, progoitrin; GRA, glucoraphanin; GAS, glucoalyssin; GNA, gluconapoleiferin; GNP, gluconapin; GBN, glucobrassicanapin; and GER, glucoerucin **(E)** Bar graph showing the amounts of four indolic GSL compounds in ‘Chiifu’ and ‘LP08’. **(F)** Pie charts showing the amounts of four indolic GSL compounds in ‘Chiifu’ and ‘LP08’. GBC, glucobrassicin; 4-HGB, 4-hydroxyglucobrassicin; 4-MTGB, 4-methoxyglucobrassicin; NGB, neoglucobrassicin.

The vegetable-type ‘Chiifu’ and oilseed-type ‘LP08’ inbred lines grown under LD conditions were harvested to quantify GSL compounds using HPLC. Twelve GSL compounds representing all three GSL groups (aliphatic, indolic, and aromatic) were successfully identified using our detection system (**Supplementary Figure S1**). Among these 12 GSLs, 7 aliphatic, 4 indolic, and 1 aromatic GSL compound were identified (**Supplementary Figure S1** and **Table S1**). The aromatic GSL compound GNT was detected at a very low level, and was therefore neglected in further analyses. Total GSL levels were apparently higher in ‘LP08’ than ‘Chiifu’ (**Figure 1A** and **Supplementary Table S1**). For example, the total GSL levels were 5,434.3 nmol/g FW in ‘LP08’ and 2,869.9 nmol/g FW in ‘Chiifu’; thus, ‘LP08’ had around double the GSL level of ‘Chiifu’ (**Figure 1B** and **Supplementary Table S1**). In the LD sample, 94.74% of GSLs in ‘LP08’ and 91.37% in ‘Chiifu’ were aliphatic, while the remaining GSLs were indolic (**Figure 1B**). While the total amount of aliphatic GSLs in ‘LP08’ was almost double that in ‘Chiifu’, the total amounts of indolic GSLs did not differ dramatically between the two inbred lines, although the difference was found to be statistically significant (**Figure 1A** and **Supplementary Table S1**). Taken together, these results indicate that, in *B. rapa* seedlings, the majority of total GSLs are aliphatic GSLs.

Among aliphatic GSLs, two compounds (GNP and GER: glucoerucin) were present at significantly higher levels in ‘LP08’ than ‘Chiifu’ (**Figure 1C**). In particular, in ‘LP08’, GNP accounted for an overwhelming proportion of aliphatic GSLs (97.5% of total aliphatic GSLs) (**Figure 1D**). These results indicate that the higher level of total GSLs in ‘LP08’ is attributable to high abundance of GNP. Meanwhile, among aliphatic GSLs in ‘Chiifu’, five compounds (PGT, GRA, GAS, GNA: gluconapoleiferin, and GBN: glucobrassicanapin) had higher levels in ‘Chiifu’ than ‘LP08’ (**Figure 1C**). Among these compounds, GRA accounted for 50.0% (1,310.5 nmol/g FW) of total aliphatic GSLs, while GNP and GBN accounted for 30.3% (793.5 nmol/g FW) and 13.2% (347.2 nmol/g FW) of total GSLs, respectively (**Figure 1D**).

Two indolic GSLs (4-methoxyglucobrassicin: 4-MTGB and NGB) had higher levels in ‘LP08’ than ‘Chiifu’, while two other GSL compounds (glucobrassicin [GBC] and 4-hydroxyglucobrassicin [4-HGB]) were present at higher levels in ‘Chiifu’ than ‘LP08’, demonstrating a dynamic compositional difference of indolic GSLs between the two lines (**Figure 1E**). In terms of total indolic GSL levels, ‘Chiifu’ and ‘LP08’ had similar amounts (241.0 and 280.5 nmol/g FW, respectively; **Figure 1F** and **Supplementary Table S1**). However, detailed analysis of the composition of indolic GSL compounds revealed differing profiles (**Figure 1F**). For example, in ‘Chiifu’ 4-HGB was dominant (49.2%) among the four measured indolic GSL compounds. Meanwhile, NGB was dominant (55.7%) among the four indolic GSLs in ‘LP08’. This indicates that ‘Chiifu’ and ‘LP08’ have differing compositional profiles for both aliphatic and indolic GSLs. Taken together, these results show that ‘LP08’ had a higher abundance of total GSLs than ‘Chiifu’, which can be attributed to greater accumulation of GNP, whereas several aliphatic GSL compounds were present in large proportions in ‘Chiifu’.

### 3.2 RNA-seq and GO analyses

**Figure 2:**
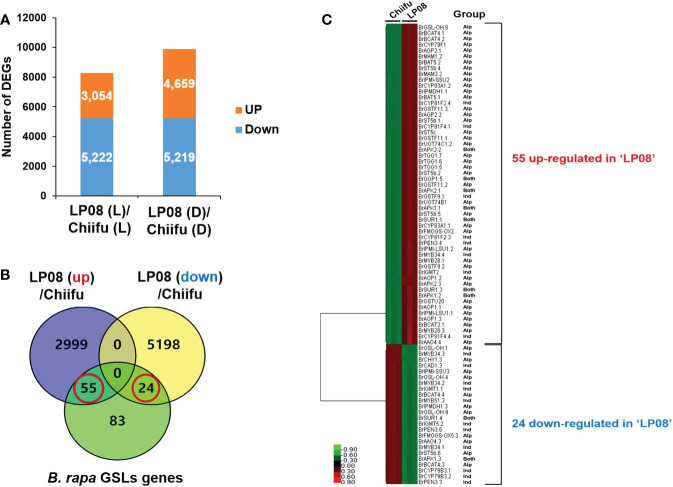
Identification of differentially expressed genes (DEGs) between ‘Chiifu’ and ‘LP08’ under light and dark conditions. **(A)** Bar graph showing the number of differentially expressed genes revealed by pairwise comparison between ‘Chiifu’ and ‘LP08’ grown under light (L) or dark (D) conditions. **(B)** Venn diagram showing 79 GSL pathway genes found among the 3,054 up-regulated and 5,222 down-regulated genes in ‘LP08’. Out of these 79 genes, 55 and 24 were up- and down-regulated, respectively, in ‘LP08’ compared to ‘Chiifu’. **(C)** Heatmap of 79 GSL pathway DEGs between ‘Chiifu’ and ‘LP08’. Alp: aliphatic GSL pathway, Ind: indolic GSL pathway.

To identify the candidate genes responsible for the observed differences in amounts and profiles of GSLs between ‘Chiifu’ and ‘LP08’, RNA-seq was performed using ‘Chiifu’ and ‘LP08’ grown under light and dark conditions. MDS and correlation heatmap analyses of RNA-seq samples showed close clustering within each sample group, indicating that RNA-seq libraries were properly generated for ‘Chiifu’ and ‘LP08’ (**Supplementary Figures S2A, B**). We isolated DEGs between the two parental lines based on comparison of pairwise samples (**Supplementary Figure S2C**). Under light conditions, 3,054 and 5,222 genes were up- and down-regulated, respectively, in ‘LP08’ compared to ‘Chiifu’. Under dark conditions, 4,659 and 5,219 genes were up- and down-regulated, respectively, in ‘LP08’ relative to ‘Chiifu’ (**Figure 2A** and **Supplementary Table S3**).

GO analysis allows for functional annotation by classifying individual genes based on their biological, cellular, and molecular functions. Thus, lists of up- and down-regulated genes in ‘LP08’ compared to ‘Chiifu’ grown under light and dark conditions were assessed with the ShinyGO analysis tool. We set 30% as an arbitrary threshold for significance. Interestingly, GO analysis of up-regulated genes in ‘LP08’ grown under light conditions revealed four categories related to GSL metabolism among the top 10 enriched categories (**Supplementary Figure S3A**). This result indicates that GSL-related metabolism is affected significantly more in ‘LP08’ compared to ‘Chiifu’. We also obtained the top 10 categories of down-regulated genes in ‘LP08’ compared to ‘Chiifu’. However, we detected no significant enrichment of GO categories above the threshold (**Supplementary Figure S3B**). These results suggest that GSL metabolism is an actively enhanced metabolic process in ‘LP08’ relative to ‘Chiifu’ grown in the light.

### 3.3 Identification of GSL pathway DEGs between ‘Chiifu’ and ‘LP08’

We performed a search using the Basic Local Alignment Search Tool (BLAST) to identify GSL metabolic genes in the *B. rapa* genome, using sequence information for *Arabidopsis* GSL pathway genes collected from The *Arabidopsis* Information Resource (TAIR) website. In total, we collected 162 GSL pathway genes (19 TFs and 143 metabolic pathway genes) from the BRAD website (**Supplementary Table S2**). Among 162 *B. rapa* GSL pathway genes, 79 were DEGs between the ‘Chiifu’ and ‘LP08’ lines under light conditions (**Supplementary Table S4**). Among 79 GSL-related genes, 55 and 24 were up- and down-regulated, respectively, in ‘LP08’ compared to ‘Chiifu’ (**Figure 2B** and **Supplementary Table S4**). Interestingly, a majority of the up-regulated genes in ‘LP08’ (49 of 55 genes; 89%) were involved in the aliphatic GSL biosynthetic pathway (**Figure 2C** and **Supplementary Table S4**). This finding is in an agreement with the result showing significantly higher amounts of aliphatic GSLs in ‘LP08’ than ‘Chiifu’ (**Figures 1A, B**). Among the 24 down-regulated genes in ‘LP08’ compared to ‘Chiifu’, both aliphatic and indolic GSL pathway genes were present in almost equal numbers (**Figure 2C**).

### 3.4 Expression of *B. rapa* MYB TFs regulating GSL biosynthesis

**Figure 3:**
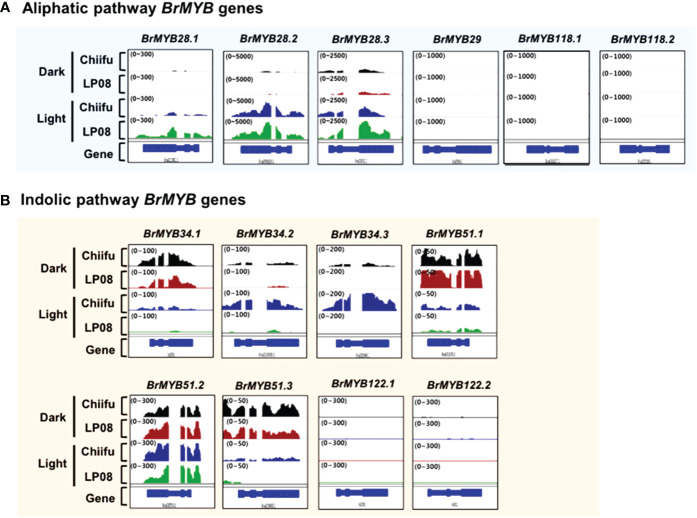
Expression profiles of *BrMYB* transcription factor **(TF)** genes between ‘Chiifu’ and ‘LP08’ grown under light and dark conditions. **(A)** Integrative genome browser (IGV) illustration of the expression profiles of six *BrMYB* genes involved in the aliphatic GSL pathway. Normalized read counts for each gene are indicated by black, brown, blue, and green colors for ‘Chiifu’ grown in the dark, ‘LP08’ grown in the dark, ‘Chiifu’ grown in the light, and ‘LP08’ grown in the light, respectively. Read coverage normalized to the total number of mapped reads is indicated in parentheses at the top left corner of each track. **(B)** IGV illustration of the expression profiles of eight *BrMYB* genes involved in the indolic GSL pathway. Normalized read counts for each gene are indicated by black, brown, blue, and green colors for ‘Chiifu’ grown in the dark, ‘LP08’ grown in the dark, ‘Chiifu’ grown in the light, and ‘LP08’ grown in the light, respectively. Read coverage, normalized using the total number of mapped reads, is indicated in parentheses at the top left corner of each track.

A subgroup of R2R3-type MYB TFs was reported to regulate GSL pathway genes. In total, 14 *B. rapa* MYB homologs (3 *BrMYB28* homologs, 1 *BrMYB29*, 3 *BrMYB34*, 3 *BrMYB51*, 2 *BrMYB118*, and 2 *BrMYB122*) were identified in BRAD (**Supplementary Table S2**). We examined the expression profiles of 14 *BrMYB* TFs between ‘Chiifu’ and ‘LP08’ grown under light and dark conditions using IGV. First, we analyzed six *BrMYBs* (*BrMYB28.1*–*3*, *BrMYB29*, and *BrMYB118.1*–*2*) involved in the aliphatic GSL pathway (**Figure 3A**). Expression levels of *BrMYB28.1* and *BrMYB28.3* were significantly higher in ‘LP08’ than ‘Chiifu’, whereas transcript levels of *BrMYB28.2* were similar between the two lines. Transcripts of *BrMYB29* and the two *BrMYB118* genes (*BrMYB118.1*–*2*) were not detected in young seedling plants grown under light or dark conditions. To validate the RNA-seq data, we conducted qRT-PCR analysis of aliphatic GSL pathway *BrMYB* genes (**Supplementary Figure S4A**). The results were similar to the RNA-seq data, confirming higher expression of *BrMYB28s* genes in ‘LP08’ than ‘Chiifu’. Therefore, higher expression of *BrMYB28.1* and *BrMYB28.3* in ‘LP08’ likely contributes to the greater accumulation of aliphatic GSLs seen in ‘LP08’ than ‘Chiifu’.

Among indolic pathway *BrMYB* TFs, the expression levels of eight *BrMYB* TFs (*BrMYB34.1*–*3*, *BrMYB51.1*–*3*, and *BrMYB122.1*–*2*) were compared between ‘Chiifu’ and ‘LP08’ under light and dark conditions (**Figure 3B**). Among these genes, the expression of *BrMYB34.1, BrMYB51.1*, and *BrMYB51.3* was strongly enhanced under dark conditions in both ‘Chiifu’ and ‘LP08’, whereas *BrMYB34.2* and *BrMYB34.3* were highly expressed under light conditions in the ‘Chiifu’ line but not the ‘LP08’ line (**Figure 3B**). While *BrMYB51.2* was expressed consistently regardless of genotype or light conditions, the two *BrMYB122* genes (*BrMYB122.1* and *BrMYB122.2*) and *BrMYB34.1* were expressed only in young seedlings under both light and dark conditions (**Figure 3B**). This suggested that individual *BrMYB* genes related to the indolic GSL pathway might regulate indolic GSL biosynthesis under certain environmental conditions or genotypes *via* diverse processes. Validation of RNA-seq data using qRT-PCR provided similar results (**Supplementary Figure S4B**). For example, *BrMYB34.1*, *BrMYB34.2*, and *BrMYB51.3* in the indolic GSL pathway were highly expressed in the dark, while *BrMYB34.3* was expressed in ‘Chiifu’ but not in ‘LP08’ (**Figure 3B** and **Supplementary Figure S4B**). Taken together, these results show that indolic GSL pathway *BrMYB* genes exhibit dynamic expression patterns in ‘Chiifu’ and ‘LP08’ under light and dark conditions, whereas aliphatic GSL pathway *BrMYB* genes are abundantly expressed in the light, particularly in the ‘LP08’ line.

Notably, the expression levels of aliphatic *BrMYB* genes were substantially higher than those of *BrMYB* genes in the indolic GSL pathway. The maximum intensity (y-axis of each IGV track view) of *BrMYB28.2* and *BrMYB28.3* was set to 5,000 and 2,500 read counts, respectively, whereas for *BrMYB51.2* and *BrMYB34.3* the values were 300 and 200, respectively. This difference indicates that aliphatic GSL pathway *BrMYBs* had relatively high expression compared to *BrMYB* genes in the indolic GSL pathway (**Figure 3**). This finding is in accordance with the level of aliphatic GSLs being substantially higher than the level of indolic GSLs in both the ‘LP08’ and ‘Chiifu’ lines.

### 3.5 Expression profiles of GSL metabolic genes

**Figure 4:**
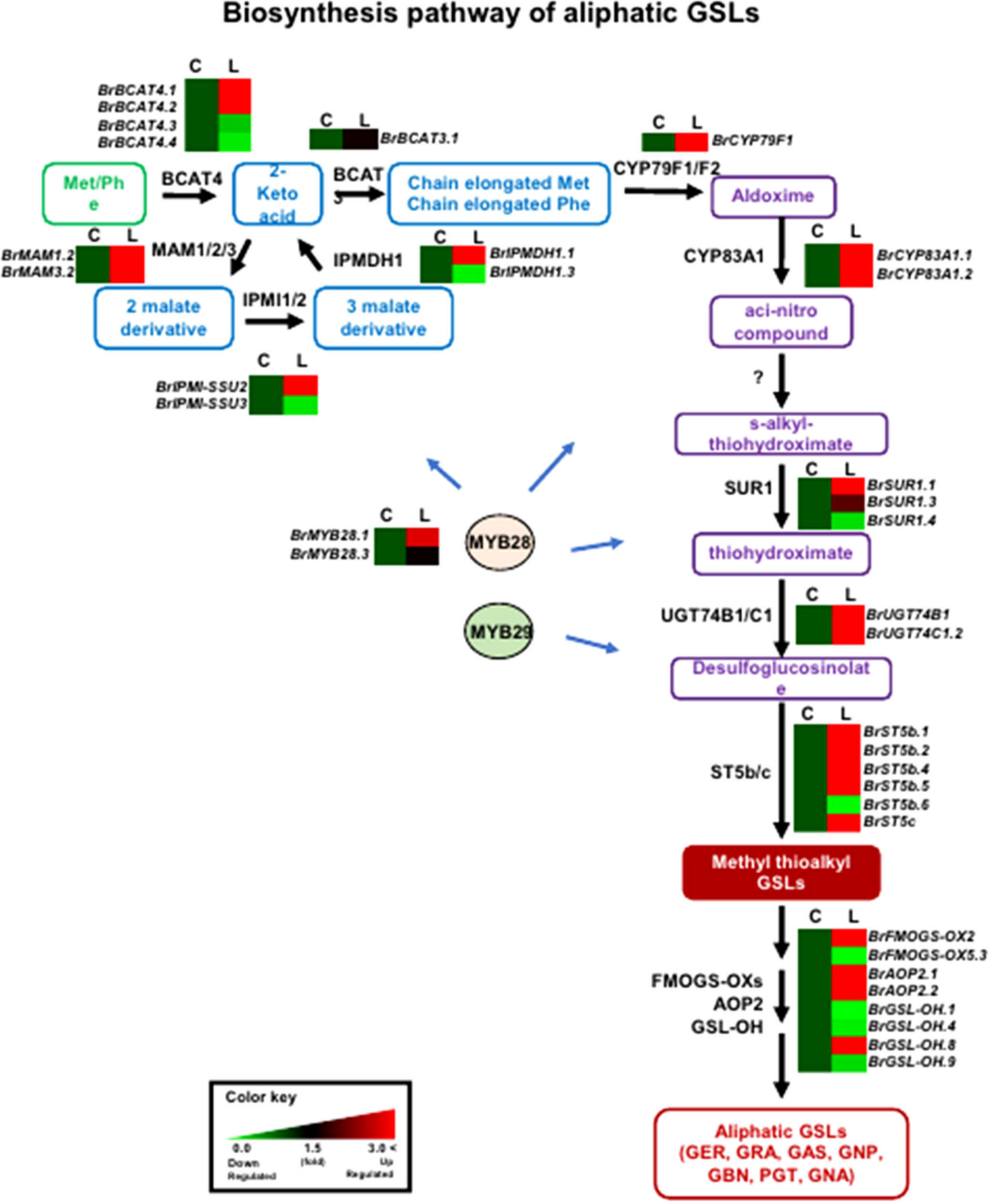
Comparison of expression profiles of GSL metabolic genes involved in aliphatic GSL biosynthesis. Schematic representation of aliphatic GSL metabolic pathways and expression patterns of aliphatic GSL pathway genes in ‘Chiifu’ and ‘LP08’. The aliphatic GSL biosynthetic pathway begins with side-chain elongation of precursor amino acids such as methionine (Met) by catalytic enzymes such as BCAT4. Black letters between paths refer to genes associated with each step. *B*. *rapa* MYB TFs, such as *BrMYB28s* (*BrMYB28.1* and *BrMYB28.3*), boost the expression of aliphatic GSL metabolic genes. Compared to ‘Chiifu’, *BrMYB28s* and downstream GSL metabolic genes are more abundantly expressed in ‘LP08’. For comparative heatmap analysis of ‘Chiifu’ and ‘LP08’, the level in ‘Chiifu’ was set to 1 and the relative level of each gene in ‘LP08’ is presented on the right side. C: Chiifu, L: LP08.

In general, genes involved in aliphatic and indolic GSL metabolism have been identified using *Arabidopsis* as a model plant (**Supplementary Table S2**). The GSL metabolic process can be grouped into five stages: ‘side-chain elongation’, ‘core structure formation’, ‘secondary modification’, ‘co-substrate’, and ‘breakdown’ (**Supplementary Table S2**). Among 143 *B. rapa* GSL metabolic genes (excluding TF genes), 72 (50%) were differentially expressed between the two lines grown in light conditions, with 55 being up-regulated and 24 down-regulated in ‘LP08’ compared to ‘Chiifu’ (**Figure 2C** and **Supplementary Figure S4**). A majority of up-regulated genes (46 of 55 in total; 84%) were involved in the aliphatic GSL pathway. Thus, it is plausible that higher expression of upstream *BrMYB* TF genes (*BrMYB28.1* and *BrMYB28.3*) in ‘LP08’ relative to ‘Chiifu’ results in up-regulation of downstream metabolic genes involved in aliphatic GSL biosynthesis (**Figure 4**).

**Figure 5:**
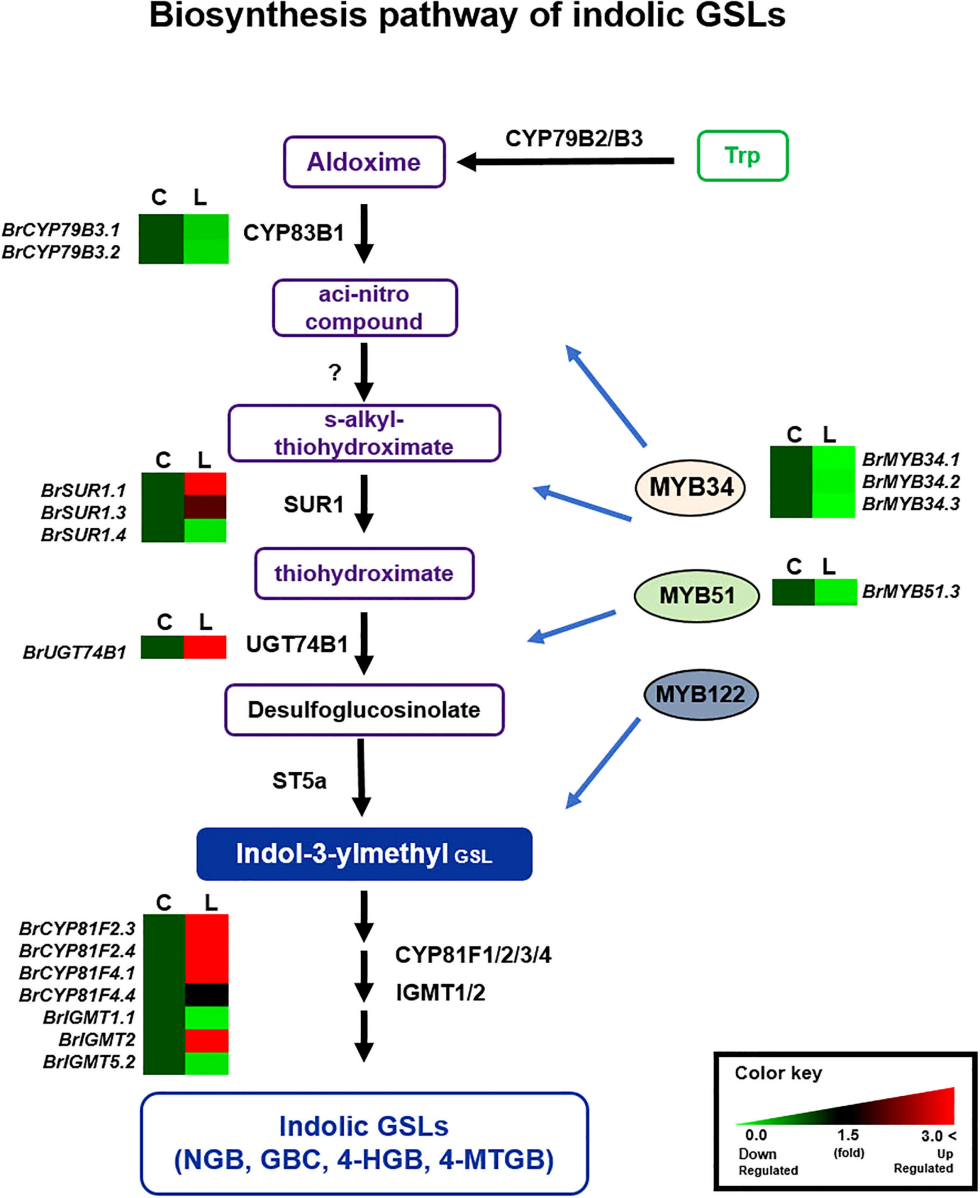
Comparison of expression profiles of GSL metabolic genes involved in indolic GSL biosynthesis. Schematic representation of indolic GSL metabolic pathways and expression patterns of indolic GSL pathway genes in ‘Chiifu’ and ‘LP08’. The indolic GSL biosynthetic pathway begins with core structure formation from precursor amino acids, such as tryptophan (Trp), by catalytic enzymes such as CYP79B2. Black letters between paths refer to genes associated with each step. *B*. *rapa* MYB TFs such as BrMYB34s, BrMYB51s, and BrMYB122 boost the expression of indolic GSL metabolic genes. Expression profiles of *BrMYB34s, BrMYB51s, BrMYB122s*, and downstream indolic GSL metabolic genes, exhibited complex differences between ‘Chiifu’ and ‘LP08’. For comparative heatmap analysis between ‘Chiifu’ and ‘LP08’, the level in ‘Chiifu’ was set to 1 and the relative level of each gene in ‘LP08’ is presented on the right side. C: Chiifu, L: LP08.

For the 24 down-regulated genes in ‘LP08’ (up-regulated in ‘Chiifu’), 11, 11 and 2 genes were related to the aliphatic, indolic, and both pathways, respectively (**Figure 5** and **Supplementary Table S4**). This result indicates that indolic GSL metabolism is more diverse and complicated in these two lines than aliphatic GSL metabolism. We reasoned that the diverse composition of aliphatic and indolic GSLs in ‘Chiifu’ and ‘LP08’ might be derived in part from the dynamic expression of GSL metabolic genes. Taken together, our findings indicate that oilseed-type *B. rapa* ‘LP08’ had higher expression of *BrMYB28* TFs and aliphatic GSL metabolic genes compared to ‘Chiifu’, resulting in greater accumulation of aliphatic GSLs in ‘LP08’. Among aliphatic GSL compounds, GNP was extraordinarily dominant. How the upregulation of *BrMYB28s* and downstream GSL metabolic genes drives specific accumulation of GNP among aliphatic GSL compounds remains unclear and requires further investigation.

**Figure 6:**
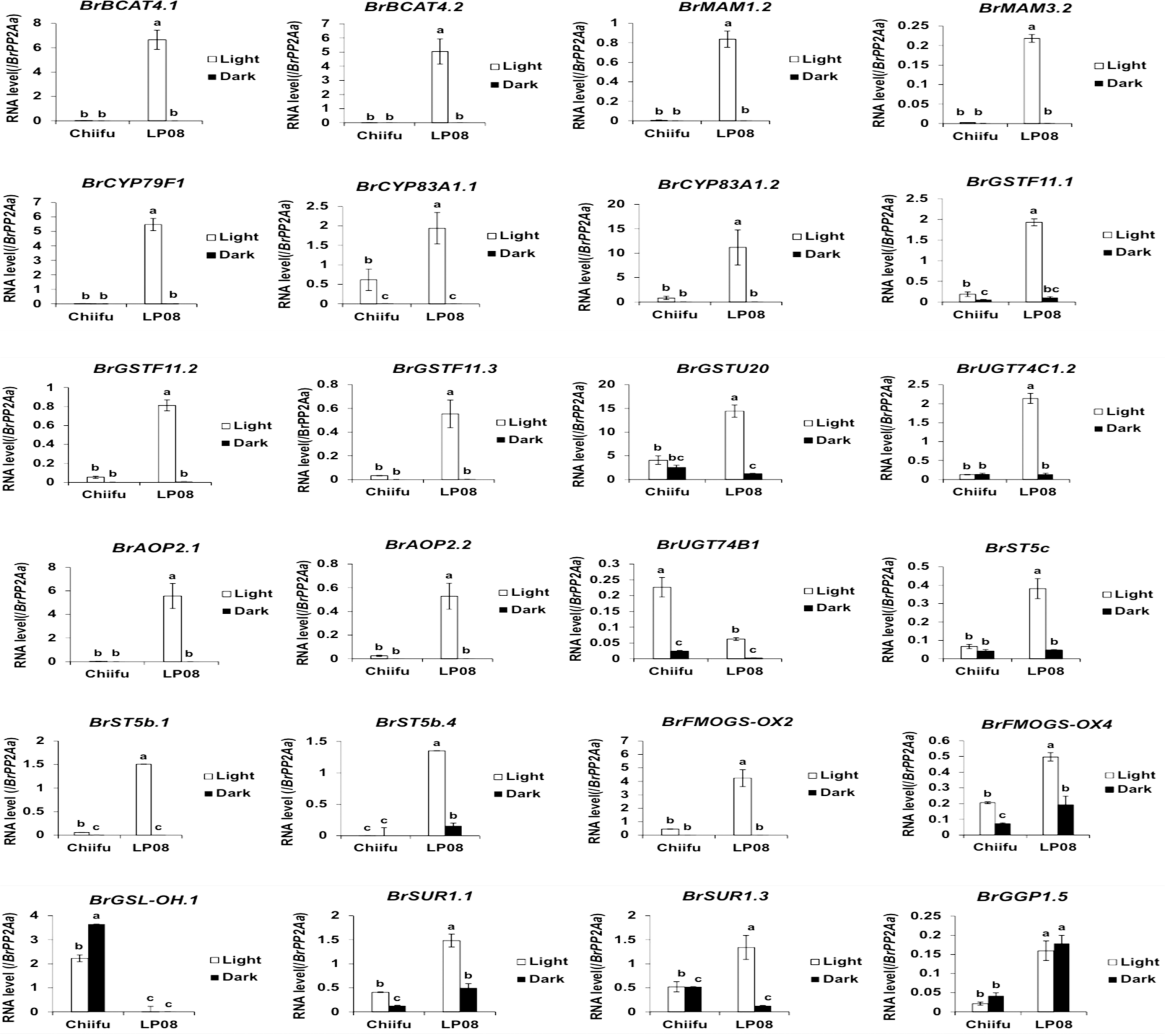
qRT-PCR analysis of 24 aliphatic GSL metabolic genes in ‘Chiifu’ and ‘LP08’ grown under light and dark conditions. Results of qRT-PCR analysis of 24 metabolic genes involved in aliphatic GSL biosynthesis. White and black bars indicate the expression level of each gene under light and dark conditions, respectively. Average values and standard deviations were calculated using the threshold cycle (Ct) values of three biological replicates. Significance was statistically determined using one-way analysis of variance (ANOVA) and Tukey’s *post-hoc* test (*p* < 0.05), and indicated with different letters above bars.

**Figure 7:**
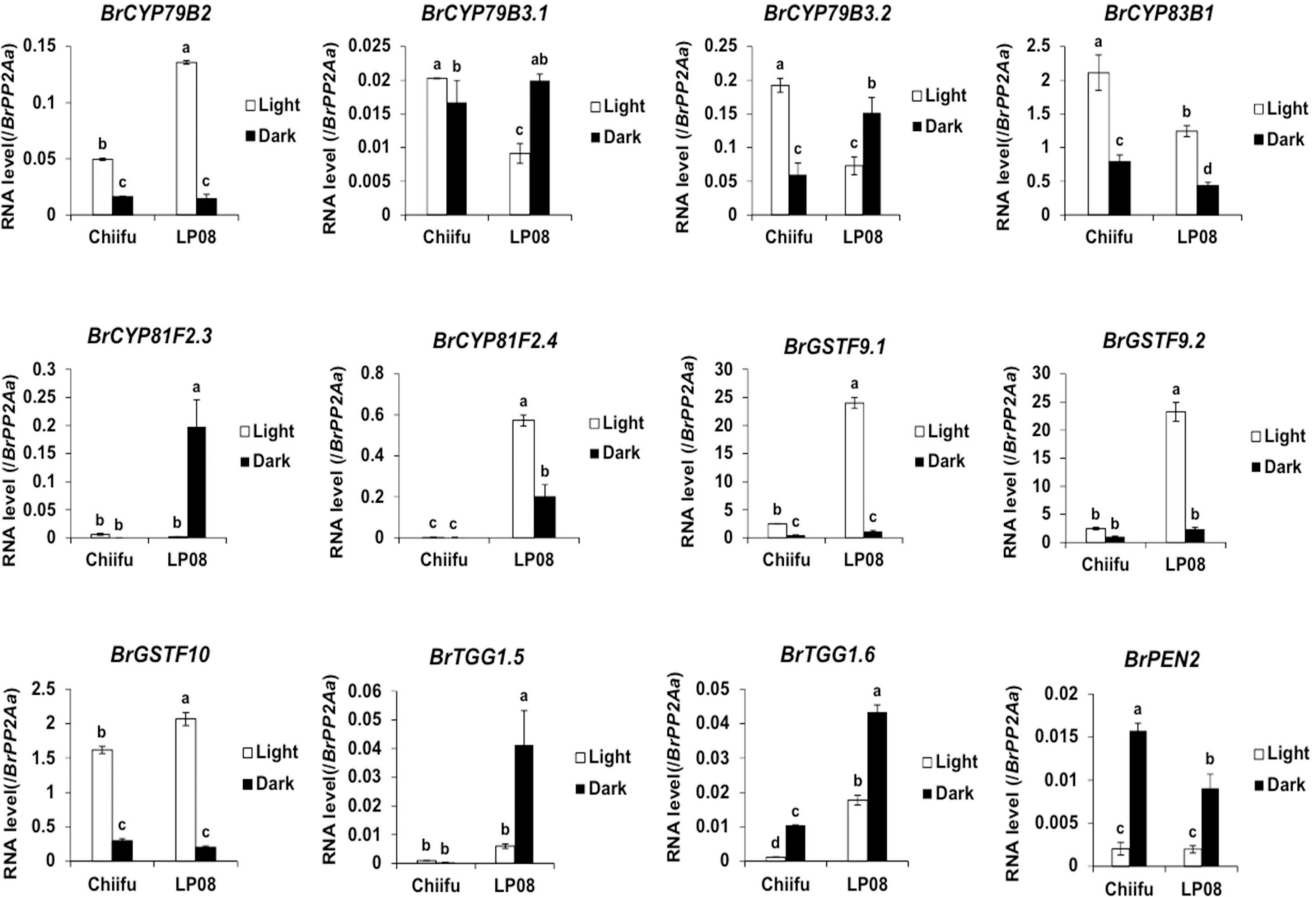
qRT-PCR analysis of 24 aliphatic GSL metabolic genes in ‘Chiifu’ and ‘LP08’ grown under light and dark conditions. Results of qRT-PCR analysis of 12 metabolic genes involved in indolic GSL biosynthesis. White and black bars indicate the expression level of each gene under light and dark conditions, respectively. Average values and standard deviations were calculated from the Ct values of three biological replicates. Significance was statistically determined using one-way ANOVA and Tukey’s *post-hoc* test (*p* < 0.05), and indicated with different letters above bars.

Validation of the RNA-seq results was performed using qRT-PCR for 36 selected genes (24 aliphatic and 12 indolic pathway genes) involved in various stages of GSL metabolism. Among the 24 metabolic genes in the aliphatic GSL pathway, 23 were more abundantly expressed in ‘LP08’ than ‘Chiifu’; the 1 exception was *BrGSL-OH.1* (**Figure 5**). Aliphatic GSL genes involved in the ‘secondary modification’ stage, such as *BrFMOGS-OX2*, *BrFMOGS-OX4*, *BrAOP2.1*, *BrAOP2.2*, *BrST5b.1*, *BrST5b.4*, and *BrST5c*, were more abundant in ‘LP08’ than ‘Chiifu’. For the *GSL-OH.1* gene, the transcript level was much higher in ‘Chiifu’ than ‘LP08’, and was also higher in the dark than light condition. *GSL-OH.1* had low expression in ‘LP08’ under both light and dark conditions, in accordance with the RNA-seq results. In addition, between the light and dark conditions, most of the tested aliphatic GSL genes were more dominant in the light than dark, confirming the results of RNA-seq. In total, 12 genes related to the indolic GSL pathway were subjected to qRT-PCR and the results were similar to the RNA-seq data, confirming that transcript patterns of indolic pathway genes differ between ‘Chiifu’ and ‘LP08’, under both light and dark conditions, more than aliphatic GSL genes (**Figures 6**, **7**). Taken together, these results indicate that the oilseed-type *B. rapa* ‘LP08’ has higher expression of GSL metabolic genes, particularly aliphatic GSL pathway genes, than the vegetable-type ‘Chiifu’ line.

### 3.6 Identification of major QTLs associated with GSL content: Results of QTL mapping

**Figure 8:**
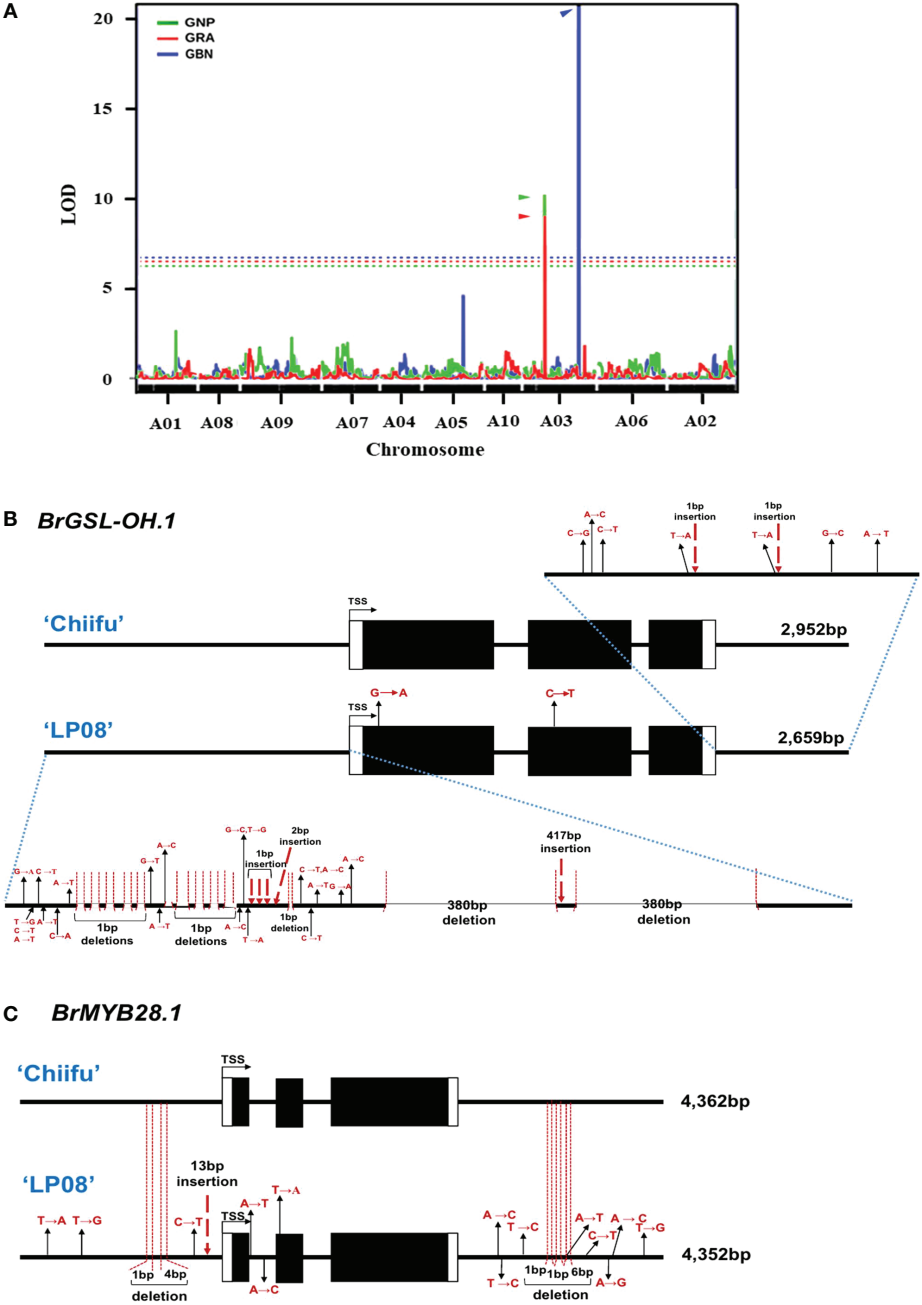
Identification of major QTL genes controlling GSL content in ‘Chiifu’ and ‘LP08’. **(A)** Results of QTL mapping of 97 F_5_ RILs and parental lines using HPLC data for GSL contents under long-day conditions. The X-axis represents the chromosomes (in order), and the Y-axis represents the limit of detection (LOD). Two peaks exceeding the LOD threshold for GRA and GNP were detected in the upper and lower arm regions for GBN in chromosome A03. The picture is made by overlapping three pictures to show the location of QTL peaks well. Green, red, and blue lines indicate the QTL peaks of GNP, GRA, and GBN, respectively and each threshold (dotted lines) is 5.454, 5.906, and 6.745. **(B)** Comparison of genomic structures of *BrGSL-OH.1* between ‘Chiifu’ and ‘LP08’. The entire 2,952- and 2,659-bp genomic sequences of *BrGSL-OH.1* were amplified through PCR from ‘Chiifu’ (upper) and ‘LP08’ (lower), respectively. Comparison of the coding region of *BrGSL-OH.1* revealed two single-base substitutions (marked with black arrows) in the first and second exon of ‘LP08’. Compared to *BrGSL-OH.1* in ‘Chiifu’, the promoter region of *BrGSL-OH.1* in ‘LP08’ had numerous single-base substitutions (marked with black arrows), along with 10 deletions (indicated with vertical dotted lines). Two large 380-bp deletions and one 417-bp insertion were identified in the promoter region of ‘LP08’. In the 3′ downstream region, seven single-base substitutions (marked with black arrows) and two single-base deletions (indicated with vertical dotted lines) were identified in ‘LP08’ compared to ‘Chiifu’. **(C)** Comparison of the genomic structures of *BrMYB28.1* between ‘Chiifu’ and ‘LP08’. The entire 4,362- and 4,352-bp genomic sequences of *BrMYB28.1* were amplified by PCR from ‘Chiifu’ (upper) and ‘LP08’ (lower), respectively. Compared to ‘Chiifu’, four single-base substitutions (marked with black arrows), one single-base deletion and one 4-base deletion (indicated with vertical dotted lines), and one 13-bp insertion (marked with red dashed arrow) were identified in the promoter region of ‘LP08’. In the coding sequence region, two single-base substitutions and one single-base substitution were detected in the first intron and second exon region, respectively, in ‘LP08’. The 3′ downstream region of *BrMYB28.1* in ‘LP08’ contained eight single-base substitutions (marked with black arrows) and three deletions (indicated with vertical dotted lines).

Previously, we developed a mapping population comprised of F_5_ 151 RILs through the crossing of ‘Chiifu’ and ‘LP08’ (Kim et al., 2022). Among the 151 F_5_ RILs, the seeds of 97 germinated successfully and grew well. These F_5_ lines, and the parental lines, were used for extraction of GSLs (**Supplementary Table S5**). The amounts of individual GSL compounds from individual F_5_ and parental lines were applied to linkage mapping based on 8,707 SNPs (**Supplementary Figure S7**). Although there are no peaks exceeding the LOD for total GSL, aliphatic GSL, and indole GSL, three QTL peaks exceeding the LOD (threshold = 5.906, 5.454, and 6.745, respectively) for GRA, GNP, and GBN were detected in chromosome A03 (**Supplementary Figure S7**; **Figure 8A**). these three peaks were corresponding to two peaks in upper arm regions (757 kb–787 kb) and the lower arm regions (approximately 1,950–2,210 kb) of total GSL and total Aliphatic GSL (**Supplementary Figure S7**). Within those regions, only two GSL pathway genes, *BrGSL-OH.1* (Bra022920) and *BrMYB28.1* (Bra012961), were located in the upper and lower arm QTL peak regions of chromosome A03, respectively. While *BrGSL-OH.1* was found at about 776 kb, *BrMYB28.1* was located at about 2,132 kb of chromosome 3. These two genes are both involved in the aliphatic GSL pathway, and no QTL candidate genes related to the indolic GSL pathway were detected in this study.

### 3.7 Comparison of genomic sequences of *BrMYB28.1* and *BrGSL-OH.1* between ‘Chiifu’ and ‘LP08’

Two QTL genes (*BrMYB28.1* and *BrGSL-OH.1*) were included in the list of DEGs between the two lines (**Figure 2C** and **Supplementary Table S4**). *BrMYB28.1*, which was located in the highest QTL peak, was more abundant in ‘LP08’ than ‘Chiifu’, whereas *BrGSL-OH.1* showed the opposite pattern, with substantially higher expression in ‘Chiifu’ than ‘LP08’ (**Figure 6**). To elucidate the molecular details underlying the differential expression of these two genes, we cloned the genomic sequences of *BrMYB28.1* and *BrGSL-OH.1* from both the ‘Chiifu’ and ‘LP08’ lines. The results confirmed that both genes underwent significant genomic “context changes” from the promoter region to the 3′ downstream region between the two inbred lines (**Figures 8B, C**). For *BrGSL-OH.1*, several mutations including large deletions and insertions were identified in the promoter region of ‘LP08’ (**Figure 8B**). In the coding region, two point mutations were identified in the first exon (G to A) and second exon (C to T). In the 3′ downstream region, seven point mutations and two single-base insertions were identified.

Multiple mutations were also detected in the *BrMYB28.1* genomic region in ‘LP08’ compared to ‘Chiifu’. Specifically, three point mutations, two deletions, and one 13-base insertion were identified in the promoter region of *BrMYB28.1* of ‘LP08’ (**Figure 8C**). In the coding region, two point mutations (A to T and A to C) were identified in the first intron and one point mutation (T to A) was found in the second exon (**Figure 8C**). In the 3′ downstream region, eight point mutations and three deletions were identified in ‘LP08’ compared to ‘Chiifu’. As shown in **Figure 3A** and **Supplementary Figure S4A**, the expression of *BrMYB28.1* was elevated in ‘LP08’ compared to ‘Chiifu’, suggesting that genomic context changes in ‘LP08’ may enhance its transcriptional activity.

## 4 Discussion

GSLs are secondary metabolites commonly synthesized in crop plants of family Brassicaceae, including important *Brassica* crops such as oilseed rape (*B. napus*), cabbage and broccoli (*B. oleracea*), and Chinese cabbage (*B. rapa*). GSLs play a major role as plant defensive compounds, and can affect the palatability and health value of edible crops (Grubb and Abel, 2006). Some GSLs and their degradation products have anti-carcinogenic and anti-oxidative activities in humans, and impart unique aromas and flavors to *Brassica* vegetables (Padilla et al., 2007). Due to their diverse roles in plant metabolism, animal nutrition, disease, and flavor, GSLs are a potential target for genetic manipulation and breeding for crop improvement. GSLs are derived from amino acids and can be divided into aliphatic (derived from Met, Leu, Ala, Ile, and Val), indolic (derived from Trp), and aromatic (derived from Phe and Tyr) classes according to their amino acid precursors (Grubb and Abel, 2006). Previous research using diverse *Brassica* subspecies reported that aliphatic GSL compounds accounted for the majority of GSLs, accounting for approximately 57−97% of the total GSL content (Kliebenstein et al., 2001; Bhandari et al., 2015; Seo et al., 2017). In accordance with a previous report (Seo et al., 2017), the total amounts of aliphatic GSLs were higher than those of indolic GSLs in this study (**Supplementary Table S1**). In addition, the amount of total GSLs (5,434.3 nmol/g) in ‘LP08’ was twice as high as in ‘Chiifu’ (2,869.9 nmol/g FW) (**Figure 1B**).

In addition to total GSL amounts, the composition of GSL compounds differed markedly between two inbred lines in terms of both aliphatic and indolic GSLs (**Figures 1D, F**). Previous studies have reported that GNP accounted for the majority of total GSLs in diverse *Brassica* species (Seo et al., 2017; Soundararajan et al., 2021). In this study, although ‘LP08’ (*B. rapa* ssp. *trilocularis*) is somewhat phenotypically different from vegetable-type *B. rapa* subspecies, GNP was the dominant aliphatic GSL compound in ‘LP08’ (**Figure 1D**). Meanwhile, ‘Chiifu’ (*B. rapa* ssp. *pekinensis*) a vegetable-type *B. rapa* line, had a diverse set of major GSLs, with three aliphatic GSLs (GRA, GNP, and GBN) exhibiting fairly similar proportions. This indicates that ‘Chiifu’ evolved to have a markedly different GSL profile from other *Brassica* subspecies. Further GSL profiling and transcriptomic analyses of diverse vegetable-type *B. rapa* plants, including ‘Chiifu’, might provide further insights into the divergent GSL profiles of *Brassica* subspecies.

In light condition, DEG analysis using RNA-seq between ‘Chiifu’ and ‘LP08’ found that 79 genes out of total 162 total GSL pathway genes were differentially expressed, showing 55 upregulated and 24 downregulated genes in ‘LP08’ in comparison to ‘Chiifu’, respectively (**Figure 2B** and **Supplementary Table S4**). Particularly, we noticed that a great portion of the upregulated genes in ‘LP08’ (49 of 55 genes; 89%) were related to the aliphatic GSL biosynthetic pathway (**Figure 2C** and **Supplementary Table S4**), explaining the reason why ‘LP08’ had significantly higher amounts of aliphatic GSLs than ‘Chiifu’ (**Figures 1A, B**). Additionally, expression levels of these 55 genes were also compared between light and dark condition, a majority of 55 genes were significantly reduced in dark samples of both ‘LP08’ and ‘Chiifu’ (**Supplementary Figure S8A**). This result suggest that aliphatic GSL biosynthesis is reduced in the absence of light, in an agreement with the previous study (Huseby et al., 2013). However, some of aliphatic GSL pathway genes including *BrAOP1.3*, *BrTGG1.6*, *BrST5b.5*, *BrAOP1.1*, and *BrAOP1.2* were rather upregulated in dark samples of both ‘Chiifu’ and ‘LP08’. Functional role of these genes in dark condition needs further investigation. Furthermore, expression pattern of 24 downregulated genes in light condition were also analyzed in the RNA-seq dataset of dark condition. Compared to 55 upregulated genes, many of 24 downregulated genes were not significantly affected in the absence of light, less sensitively affected in the dark condition (**Supplementary Figure S8B**). Taken together, these data indicate that light-mediated signaling might play a positive effect on GSL biosynthesis pathway, particularly aliphatic GSL pathway in *B. rapa*.

Numerous environmental cues, including photoperiod (i.e. circadian rhythm) and other endogenous factors (i.e. phytohormones), affect GSL biosynthesis *via* modulation of GSL pathway genes (Bohinc and Trdan, 2012). Recently, we reported that a circadian clock component, *BrGI* (*B. rapa GIGANTEA*), is involved in the regulation of GSL biosynthesis in *B. rapa via* transcriptional modulation of GSL pathway genes (Kim et al., 2021). Crosstalk among diverse circadian components to coordinate daily GSL biosynthesis in *B. rapa* plants is an interesting topic for future research.

In QTL mapping, the second LOD peak was positioned around *BrGSL-OH.1* (Bra022920) (**Figure 8A**). Genomic sequence comparison betweeen ‘Chiifu’ and ‘LP08’ revealed that *BrGSL-OH.1* contained considerable mutations in ‘LP08’ compared to ‘Chiifu’. For instance, large deletions and insertions in the promoter region, two point mutations like one in the first exon (G to A) and the other in the second exon (C to T), and seven point mutations and two single-base insertions in the 3′ downstream region (**Figure 8B**). Low expression of *BrGSL-OH.1* in ‘LP08’ might be attributed to severe mutational events, including large deletions and insertions in the promoter region of *BrGSL-OH.1*. We reasoned that these genomic mutations might result in the loss of DNA elements required for active transcription of *BrGSL-OH.1*. *Arabidopsis GSL-OH* (Arabidopsis gene ID: AT2G25450) encodes a 2-oxoacid-dependent dioxygenase involved in the production of the aliphatic GSL compound PGT (Hansen et al., 2008). PGT has biological functions including toxicity against the nematode *Caenorhabditis elegans*, inhibition of seed germination, induction of goiter disease in mammals, and bitter taste in plants of the genus *Brassica*. In this study, the expression of *BrGSL-OH.1* was significantly higher in ‘Chiifu’ than ‘LP08’, such that ‘Chiifu’ is expected to have more PGT than ‘LP08’. The amount of PGT was higher in ‘Chiifu’ (30.8 nmol/g FW) than ‘LP08’ (21.4 nmol/g FW) (**Supplementary Table S1**). However, considering the dramatic up-regulation of *BrGSL-OH.1* in ‘Chiifu’, the quantitative difference between the two lines was subtle. One explanation for this is that the *B. rapa* genome may contain functionally redundant *BrGSL-OH* homologs. A BLAST search using the *Arabidopsis* GSL-OH sequence indicated that the *B. rapa* genome contains nine *B. rapa GSL-OH* homologs (named *BrGSL-OH.1*–*9*) (**Supplementary Table S2**). Among the nine homologs of *BrGSL-OH.1* to *BrGSL-OH.9*, four were differentially expressed between the two lines (**Figure 2C** and **Supplementary Table S4**). Three *BrGSL-OH* homologs (*BrGSL-OH.1*, *BrGSL-OH.4*, and *BrGSL-OH.9*) were expressed at higher levels in ‘Chiifu’ than ‘LP08’, whereas *BrGSL-OH.8* was more abundant in ‘LP08’ than ‘Chiifu’. Therefore, the dynamic expression of multiple *BrGSL-OH* homologs in *B. rapa* may contribute to the moderate difference in PGT contents between the two inbred lines.

The highest LOD peak on the SNP-based linkage map was located in the lower arm region of chromosome A03 in the genomic region containing *BrMYB28.1* (**Figure 8A**). In *Arabidopsis*, a subgroup of MYB family TFs (*MYB28*, *MYB29*, and *MYB76*) have been reported to regulate the biosynthesis of aliphatic GSLs (Hirai et al., 2007; Gigolashvili et al., 2008; Sønderby et al., 2010a). In the *B. rapa* genome, three *MYB28* homologs [named *BrMYB28.1* (Bra012961), *BrMYB28.2* (Bra035929), *BrMYB28.3* (Bra029311)] and one *MYB29* homolog (*BrMYB29*, Bra005949) were identified (**Supplementary Table S2**). However, no *MYB76* homolog was found in the *B. rapa* genome. In this study, as well as *BrMYB28.1*, the expression of *BrMYB28.3* was also higher in ‘LP08’ than ‘Chiifu’, whereas *BrMYB28.2* had similar expression levels between the two lines. Another *BrMYB* genes, *BrMYB29* and *BrMYB118.1/2* were not expressed in our test irrespective of light condition (**Figure 3A**), suggesting that the *BrMYB29* and *BrMYB118.1/2* TFs do not contribute to aliphatic GSL biosynthesis, at least in seedling plants. *BrMYB29* and *BrMYB118s* may function in other developmental stages or under specific stress conditions. This hypothesis requires further investigation.

*BrMYB28.1* was previously reported to have the highest transcript level among the three *BrMYB28* homologs in various organs of *B. rapa* (Seo et al., 2016). According to our RNA-seq results, however, *BrMYB28.1* expression was moderate compared to *BrMYB28.2* and *BrMYB28.3*. This discrepancy might result from differences in the growth conditions or developmental status of *B. rapa* plants. A previous study reported that overexpression of *BrMYB28.1* resulted in elevated levels of aliphatic GSL compounds, indicating that BrMYB28.1 acts as a positive regulator of the GSL biosynthetic process in *B. rapa* plants, similar to the model plant *Arabidopsis* (Seo et al., 2016).

In this study, the expression of *BrMYB28.1* was higher in ‘LP08’ than ‘Chiifu’. Regarding this observation, we reasoned that mutations in the promoter region of *BrMYB28.1* in ‘LP08’ may affect a DNA element required for the recruitment of a transcriptional repressor, leading to de-repression of the *BrMYB28.1* transcript level. Alternatively, mutations such as a 13-base insertion in the promoter region of *BrMYB28.1* may create a genomic context promoting the recruitment of transcriptional activators. These possibilities require further studies, including a promoter assay and complementation analysis to clarify the differences in profiles of GSL compounds between ‘Chiifu’ and ‘LP08’. As a result of higher expression of *BrMYB28.1* in ‘LP08’, transcript levels of downstream GSL metabolic genes including *BrBCAT4.1/2*, *BrCYP79F1*, *BrCYP83.1/2*, *BrSUR1a*, *BrFMO GS-OX2*, and *BrAOP2.1/2* were also elevated in ‘LP08’ compared to ‘Chiifu’ (**Figures 4**, **6**). Therefore, elevated expression of *BrMYB28.1* and its downstream genes likely greatly enhances the accumulation of GNP in ‘LP08’. How elevated expression of *BrMYB28.1* and its downstream GSL metabolic genes leads to the overwhelming accumulation of GNP in ‘LP08’ remains unclear. Further analysis is needed to reveal the functional role of *BrMYB28.1* in the accumulation of GNP among aliphatic GSL compounds in *B. rapa* ‘LP08’ plants.

**Figure 9:**
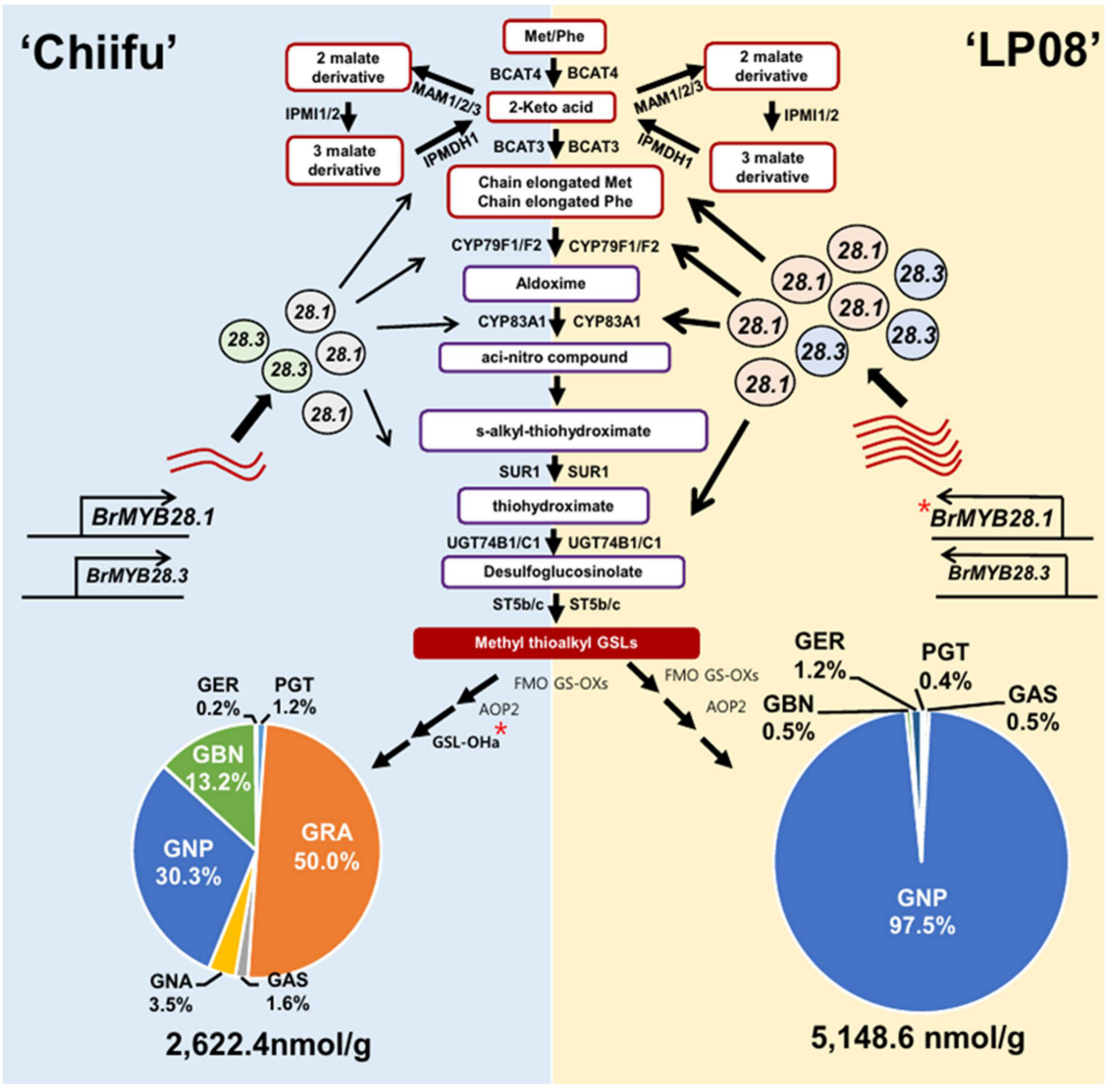
Schematic of the molecular mechanisms underlying the differing GSL profiles between vegetable-type ‘Chiifu’ and oilseed-type ‘LP08’. The aliphatic GSL biosynthetic pathways of ‘Chiifu’ (left) and ‘LP08’ (right) are represented on blue and yellow backgrounds, respectively. Three BrMYB28 homologs play a positive role in the transcription of downstream GSL metabolic genes. Compared to ‘Chiifu’, ‘LP08’ had higher expression of *BrMYB28.1* (as well as *BrMYB28.3*), which stimulated downstream GSL metabolic genes. As a result, total amounts of aliphatic GSLs were significantly higher in ‘LP08’ than ‘Chiifu’. In addition, *GSL-OH.1* was uniquely expressed in ‘Chiifu’ and not ‘LP08’, which may contribute to the differing compositional profiles of aliphatic GSLs between these two lines. ‘Chiifu’ has three major aliphatic GSL compounds accounting for large portions of its total aliphatic GSLs, whereas ‘LP08’ possessed a single GSL compound, GNP, accounting for 97.5% of the total aliphatic GSLs. Pie charts are presented as mean ± standard deviation **(SD)** (n = 3). Red asterisks (*) indicate two genes identified as major QTL candidates. PGT: progoitrin; GRE: glucoraphenin; GER: glucoerucin, GBN: glucobrassicanapin, GRA: glucoraphanin, GAS: glucoalyssin, GNP: gluconapin.

Based on the observations in this study, a conceptual model was proposed to describe the differing profiles of aliphatic GSLs between vegetable-type ‘Chiifu’ and oilseed-type ‘LP08’ (**Figure 9**). Precursor amino acids, such as Met and Phe, undergo a series of chemical modifications involving numerous enzymes related to GSL metabolism, including side chain elongation, core structure formation, and secondary modification. The *B. rapa* MYB genes *BrMYB28.1* and *BrMYB28.3* stimulate the expression of these GSL metabolic genes. Expression levels of *BrMYB28.1* and *BrMYB28.3* were higher in ‘LP08’ than ‘Chiifu’, resulting in higher expression of GSL metabolic genes related to aliphatic GSL biosynthesis in ‘LP08’. In ‘Chiifu’, the QTL gene GSL-OH.1 was more abundant than in ‘LP08’, possibly causing differences in their profiles of aliphatic GSL compounds (abundant GNP, GRA, and GBN in ‘Chiifu’ vs. dominance by GNP in ‘LP08’).

## Data availability statement

The datasets presented in this study can be found in online repositories. The names of the repository/repositories and accession number(s) can be found in the article/**Supplementary Material**.

## Author contributions

JK and D-HK planned the experiments. JK, HK and HM prepared all plant materials. HM performed the molecular experiments. DC, SL, and AN performed the HPLC analysis. JK, HK, N-SK, and JJ developed the mapping population and performed the QTL analysis. HM and D-HK analyzed the RNA-seq data. HM, JK, and D-HK wrote the draft. D-HK revised and finalized the manuscript. All authors contributed to the article and approved the submitted version.

## Supplementary material

Supplementary Figure 1 UHPLC chromatogram of GSLs extracted from seedlings of ‘LP08’ (upper) and ‘Chiifu’ (bottom). 1, progoitrin (PGT); 2, glucoraphanin (GRA); 3, glucoalyssin (GAS); 4, gluconapoleiferin (GNA); 5, gluconapin (GNP); 6, 4-hydroxyglucobrassicin (4-HGB); 7, glucobrassicanapin (GBN); 8, glucoerucin (GER); 9, glucobrassicin (GBC); 10, 4-methoxyglucobrassicin (4-MTGB); 11, neoglucobrassicin (NGB); 12, gluconasturtiin (GNT).

Supplementary Figure 2 Identification of differentially expressed genes (DEGs) between ‘Chiifu’ and ‘LP08’. **(A)** Multi-dimensional Scaling (MDS) plot showing that samples within a group had similar gene expression profiles and significant differences existed among groups. CL: Chiifu grown in the light; CD: Chiifu grown in the dark; LL: LP08 grown in the light; and LD: LP08 grown in the dark. Sample symbols are followed by the number of biological replicates (1–3). **(B)** Heatmap of the Pearson correlation matrix between samples used for pairwise comparisons. The color key was adjusted based on the log2-centered values to highlight differences. Dendrograms showing distances between repeated samples. **(C)** MA plot of DEGs between ‘Chiifu’ and ‘LP08’ grown under light (left) and dark (right) conditions. The X-axis represents average expression, logCPM (log_2_-transformed counts per minute), and the Y-axis represents log_2_-transformed fold-change values. Red dots indicate DEGs between ‘Chiifu’ and ‘LP08’. Black dots indicate non-DEGs.

Supplementary Figure 3 Gene Ontology (GO) analysis of up- and down-regulated genes between ‘Chiifu’ and ‘LP08’ grown in the light condition. Top 10 functional categories of up-regulated (left) and down-regulated genes (right) in ‘LP08’ compared to ‘Chiifu’. Categories related to the glucosinolates biosynthesis are shown in red, and red asterisks indicate significant categories. Y-axis indicate the percentage of detected category genes/total category genes which is automatically calculated by ShinyGO (v0.76) analysis tool.

Supplementary Figure 4 qRT-PCR analysis of two differentially expressed *BrMYB* genes between ‘Chiifu’ and ‘LP08’ grown under light and dark conditions. **(A)** Results of qRT-PCR analysis of two *BrMYB28s* (*BrMYB28.1* and *BrMYB28.3*) that exhibited differential expression between ‘Chiifu’ and ‘LP08’. White and black bars indicate the expression levels of each gene under light and dark conditions, respectively. Average values and standard deviations were calculated from the Ct values of three biological replicates. **(B)** Results of qRT-PCR analysis of three *BrMYB34s* (*BrMYB34.1*–*34.3*) and a *BrMYB51.1* that exhibited differential expression between ‘Chiifu’ and ‘LP08’. White and black bars indicate the expression levels of each gene under light and dark conditions, respectively. **(A, B)** Average values and standard deviations were calculated from the Ct values of three biological replicates. One-way ANOVA and Tukey’s *post-hoc* test (*p* < 0.05) were used to determine statistically significant differences. Significance is represented by different letters above bars.

Supplementary Figure 5 Integrative genome browser (IGV) illustration of the expression profiles of total 42 aliphatic GSL biosynthetic genes differentially expressed between ‘Chiifu’ and ‘LP08’ grown under light and dark conditions. **(A)** IGV illustration of the expression profiles of 15 genes involved in the ‘side-chain elongation’ stage of aliphatic GSL biosynthesis. Normalized read counts for each gene are indicated by black, blue, brown, and green colors for ‘Chiifu’ grown in the dark, ‘LP08’ grown in the dark, ‘Chiifu’ grown in the light, and ‘LP08’ grown in the light, respectively. Read coverage normalized to the total number of mapped reads is indicated in parentheses at the top left corner of each track. **(B)** IGV illustration of the expression profiles of 13 genes involved in the ‘core structure formation’ stage of aliphatic GSL biosynthesis. Normalized read counts for each gene are indicated by black, blue, brown, and green colors for ‘Chiifu’ grown in the dark, ‘LP08’ grown in the dark, ‘Chiifu’ grown in the light, and ‘LP08’ grown in the light, respectively. Read coverage normalized to the total number of mapped reads is indicated in parentheses at the top left corner of each track. **(C)** IGV illustration of the expression profiles of 11 genes involved in the ‘secondary modification’ stage of aliphatic GSL biosynthesis. Normalized read counts for each gene are indicated by black, blue, brown, and green colors for ‘Chiifu’ grown in the dark, ‘LP08’ grown in the dark, ‘Chiifu’ grown in the light, and ‘LP08’ grown in the light, respectively. Read coverage normalized to the total number of mapped reads is indicated in parentheses at the top left corner of each track. **(D)** IGV illustration of the expression profiles of three genes involved in the ‘breakdown’ stage of aliphatic GSL catabolism. Normalized read counts for each gene are indicated by black, blue, brown, and green colors for ‘Chiifu’ grown in the dark, ‘LP08’ grown in the dark, ‘Chiifu’ grown in the light, and ‘LP08’ grown in the light, respectively. Read coverage normalized to the total number of mapped reads is indicated in parentheses at the top left corner of each track.

Supplementary Figure 6 Integrative genome browser (IGV) illustration of the expression profiles of 19 total indolic GSL biosynthetic genes between ‘Chiifu’ and ‘LP08’ grown under light and dark conditions. **(A)** IGV illustration of the expression profiles of five genes involved in the ‘core structure formation’ stage of indolic GSL biosynthesis. Normalized read counts for each gene are indicated by black, brown, blue, and green colors for ‘Chiifu’ grown in the dark, ‘LP08’ grown in the dark, ‘Chiifu’ grown in the light, and ‘LP08’ grown in the light, respectively. Read coverage normalized to the total number of mapped reads is indicated in parentheses at the top left corner of each track. **(B)** IGV illustration of the expression profiles of six genes commonly involved in the ‘core structure formation’ stages of both aliphatic and indolic GSL biosynthesis. Normalized read counts for each gene are indicated by black, brown, blue, and green colors for ‘Chiifu’ grown in the dark, ‘LP08’ grown in the dark, ‘Chiifu’ grown in the light, and ‘LP08’ grown in the light, respectively. Read coverage normalized to the total number of mapped reads is indicated in parentheses at the top left corner of each track. **(C)** IGV illustration of the expression profiles of four genes involved in the ‘secondary modification’ stage of indolic GSL biosynthesis. Normalized read counts for each gene are indicated by black, blue, brown, and green colors for ‘Chiifu’ grown in the dark, ‘LP08’ grown in the dark, ‘Chiifu’ grown in the light, and ‘LP08’ grown in the light, respectively. Read coverage normalized to the total number of mapped reads is indicated in parentheses at the top left corner of each track. **(D)** IGV illustration of the expression profiles of four genes involved in the ‘breakdown’ stage of indolic GSL catabolism. Normalized read counts for each gene are indicated by black, blue, brown, and green colors for ‘Chiifu’ grown in the dark, ‘LP08’ grown in the dark, ‘Chiifu’ grown in the light, and ‘LP08’ grown in the light, respectively. Read coverage normalized to the total number of mapped reads is indicated in parentheses at the top left corner of each track.

Supplementary Figure 7 Results of QTL mapping of 97 F_5_ RILs and parental lines using HPLC data for GSL contents under long-day conditions. **(A)** Results of QTL mapping for total GSLs, total indolic GSLs, and total aliphatic GSL. **(B)** Results of QTL mapping for individual aliphatic GSL compounds. Normalized read counts for each gene are indicated by black, brown, blue, and green colors for ‘Chiifu’ X-axis represents the chromosomes (in order), and the Y-axis represents the limit of detection (LOD). AlP and Ind means aliphatic GSL and indolic GSL. The red and blue asterisks indicate peaks at the same position, respectively.

Supplementary Figure 8 Expression profile of 77 DEGs (55 up- and 24 down-regulated genes under light and dark condition. **(A)** Expression heatmap of 55 ‘LP08’-upregulated GSL pathway genes under light and dark condition. A majority of 55 ‘LP08’ up-regulated genes were significantly downregulated in both ‘Chiifu’ and ‘LP08’ dark samples. Downregulated genes in dark condition were indicated with right side blue vertical bar. Upregulated genes in the dark condition were indicated with right side red vertical bar. **(B)** Expression heatmap of 24 ‘LP08’-downregulated GSL pathway genes under light and dark condition. Many of 24 ‘LP08’-downregulated genes were not significantly affected in the absence of light, less sensitively affected in the dark condition when compared to the expression profile of 55 ‘LP08’ up-regulated genes.
