# Supplementary figures and images for: Transcriptome and QTL mapping analyses of major QTL genes controlling glucosinolate contents in vegetable- and oilseed-type Brassica rapa plants
Source: Front Plant Sci. 2023 Jan 18;13:1067508. doi: 10.3389/fpls.2022.1067508 (PMC9891538; doi:10.3389/fpls.2022.1067508)

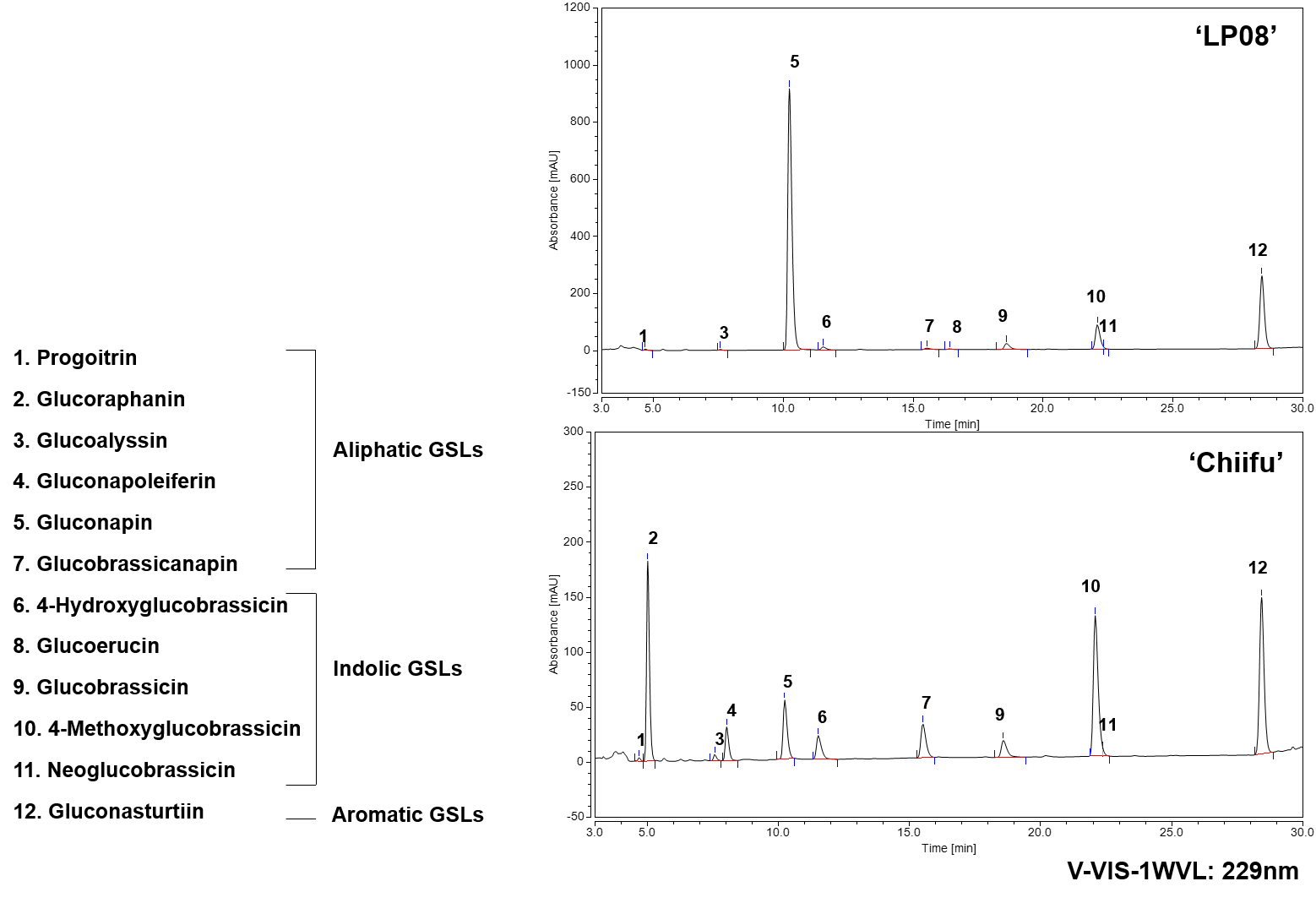

Supplement: Supplementary Figure 1 — UHPLC chromatogram of GSLs extracted from seedlings of ‘LP08’ (upper) and ‘Chiifu’ (bottom). 1, progoitrin (PGT); 2, glucoraphanin (GRA); 3, glucoalyssin (GAS); 4, gluconapoleiferin (GNA); 5, gluconapin (GNP); 6, 4-hydroxyglucobrassicin (4-HGB); 7, glucobrassicanapin (GBN); 8, glucoerucin (GER); 9, glucobrassicin (GBC); 10, 4-methoxyglucobrassicin (4-MTGB); 11, neoglucobrassicin (NGB); 12, gluconasturtiin (GNT). [file DataSheet_1.zip › Supplementary figure S/Supp Fig. S1.tif]

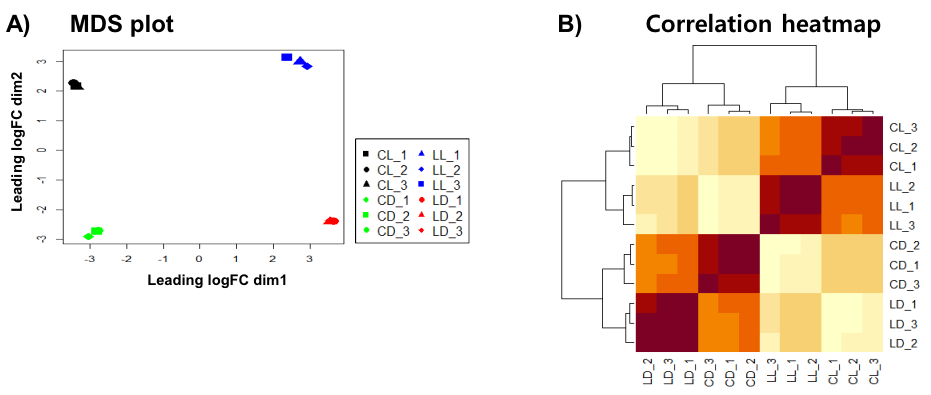

Supplement: Supplementary Figure 1 — UHPLC chromatogram of GSLs extracted from seedlings of ‘LP08’ (upper) and ‘Chiifu’ (bottom). 1, progoitrin (PGT); 2, glucoraphanin (GRA); 3, glucoalyssin (GAS); 4, gluconapoleiferin (GNA); 5, gluconapin (GNP); 6, 4-hydroxyglucobrassicin (4-HGB); 7, glucobrassicanapin (GBN); 8, glucoerucin (GER); 9, glucobrassicin (GBC); 10, 4-methoxyglucobrassicin (4-MTGB); 11, neoglucobrassicin (NGB); 12, gluconasturtiin (GNT). [file DataSheet_1.zip › Supplementary figure S/Supp Fig. S2AB.tif]

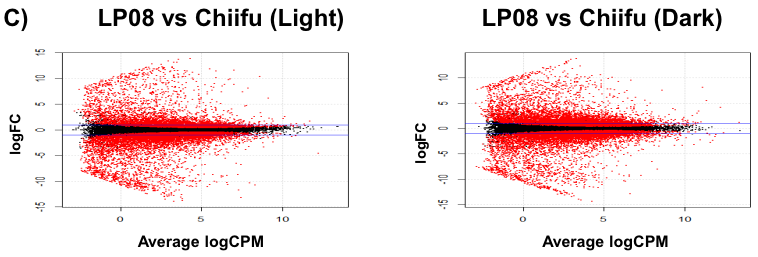

Supplement: Supplementary Figure 1 — UHPLC chromatogram of GSLs extracted from seedlings of ‘LP08’ (upper) and ‘Chiifu’ (bottom). 1, progoitrin (PGT); 2, glucoraphanin (GRA); 3, glucoalyssin (GAS); 4, gluconapoleiferin (GNA); 5, gluconapin (GNP); 6, 4-hydroxyglucobrassicin (4-HGB); 7, glucobrassicanapin (GBN); 8, glucoerucin (GER); 9, glucobrassicin (GBC); 10, 4-methoxyglucobrassicin (4-MTGB); 11, neoglucobrassicin (NGB); 12, gluconasturtiin (GNT). [file DataSheet_1.zip › Supplementary figure S/Supp Fig. S2C.tif]

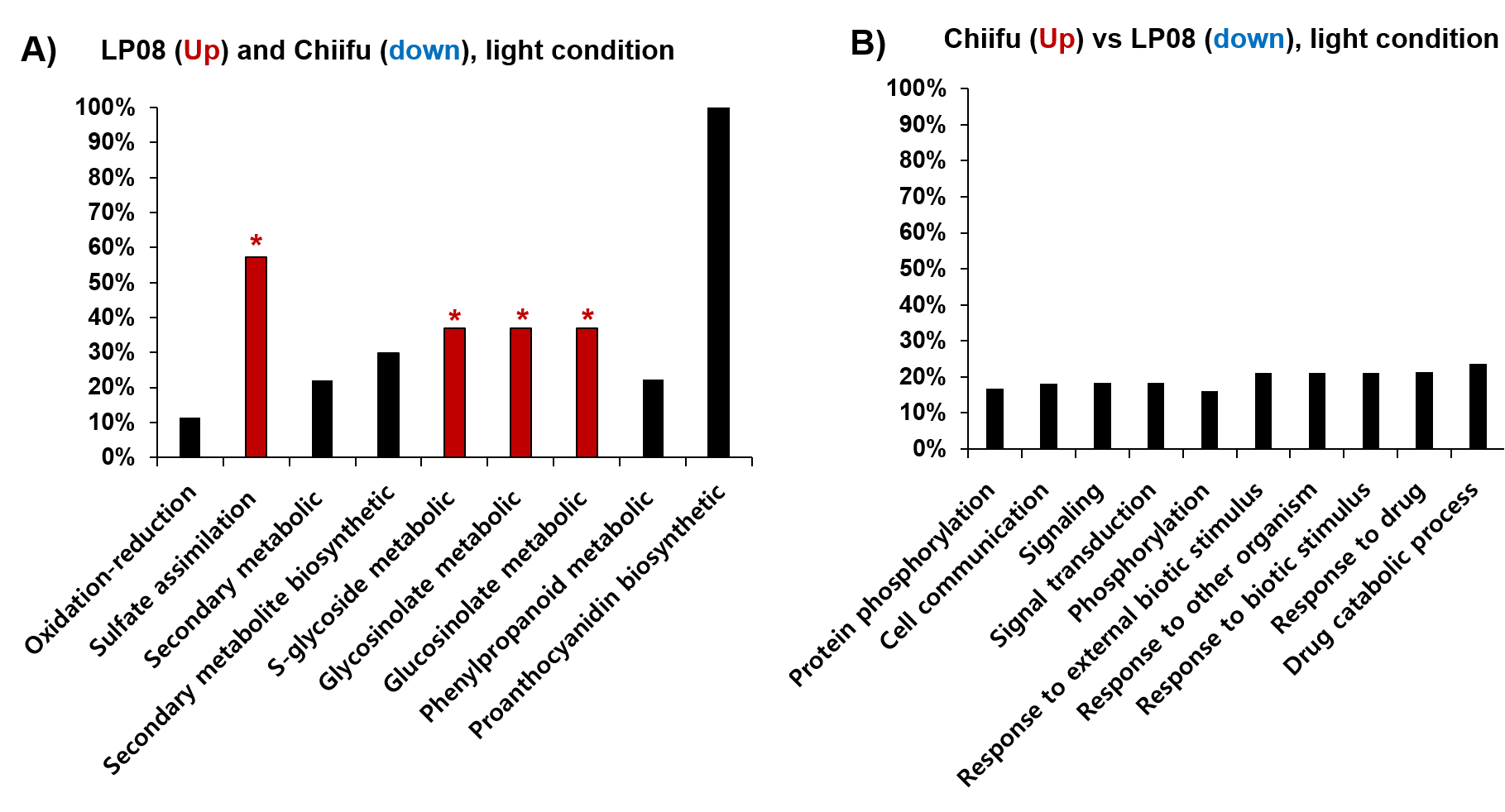

Supplement: Supplementary Figure 1 — UHPLC chromatogram of GSLs extracted from seedlings of ‘LP08’ (upper) and ‘Chiifu’ (bottom). 1, progoitrin (PGT); 2, glucoraphanin (GRA); 3, glucoalyssin (GAS); 4, gluconapoleiferin (GNA); 5, gluconapin (GNP); 6, 4-hydroxyglucobrassicin (4-HGB); 7, glucobrassicanapin (GBN); 8, glucoerucin (GER); 9, glucobrassicin (GBC); 10, 4-methoxyglucobrassicin (4-MTGB); 11, neoglucobrassicin (NGB); 12, gluconasturtiin (GNT). [file DataSheet_1.zip › Supplementary figure S/Supp Fig. S3 GO.tif]

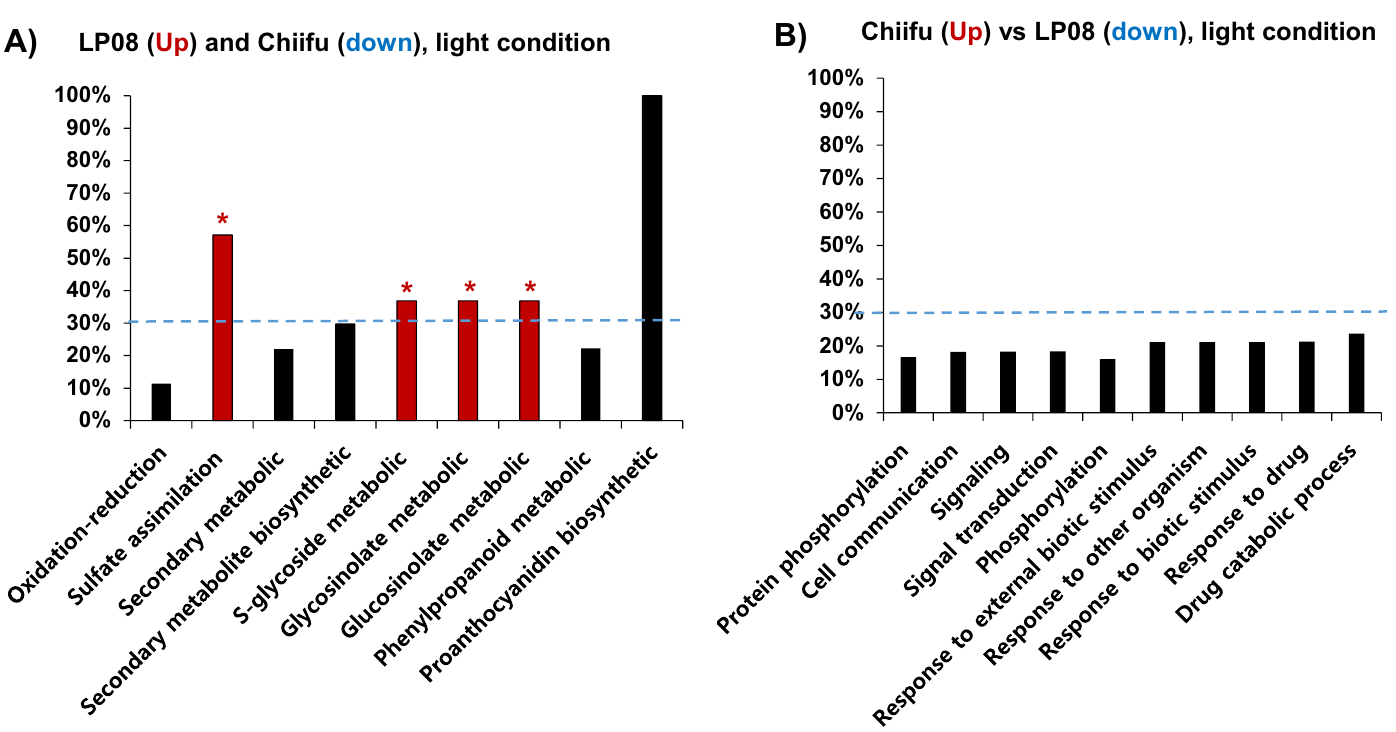

Supplement: Supplementary Figure 1 — UHPLC chromatogram of GSLs extracted from seedlings of ‘LP08’ (upper) and ‘Chiifu’ (bottom). 1, progoitrin (PGT); 2, glucoraphanin (GRA); 3, glucoalyssin (GAS); 4, gluconapoleiferin (GNA); 5, gluconapin (GNP); 6, 4-hydroxyglucobrassicin (4-HGB); 7, glucobrassicanapin (GBN); 8, glucoerucin (GER); 9, glucobrassicin (GBC); 10, 4-methoxyglucobrassicin (4-MTGB); 11, neoglucobrassicin (NGB); 12, gluconasturtiin (GNT). [file DataSheet_1.zip › Supplementary figure S/Supp Fig. S3AB.tif]

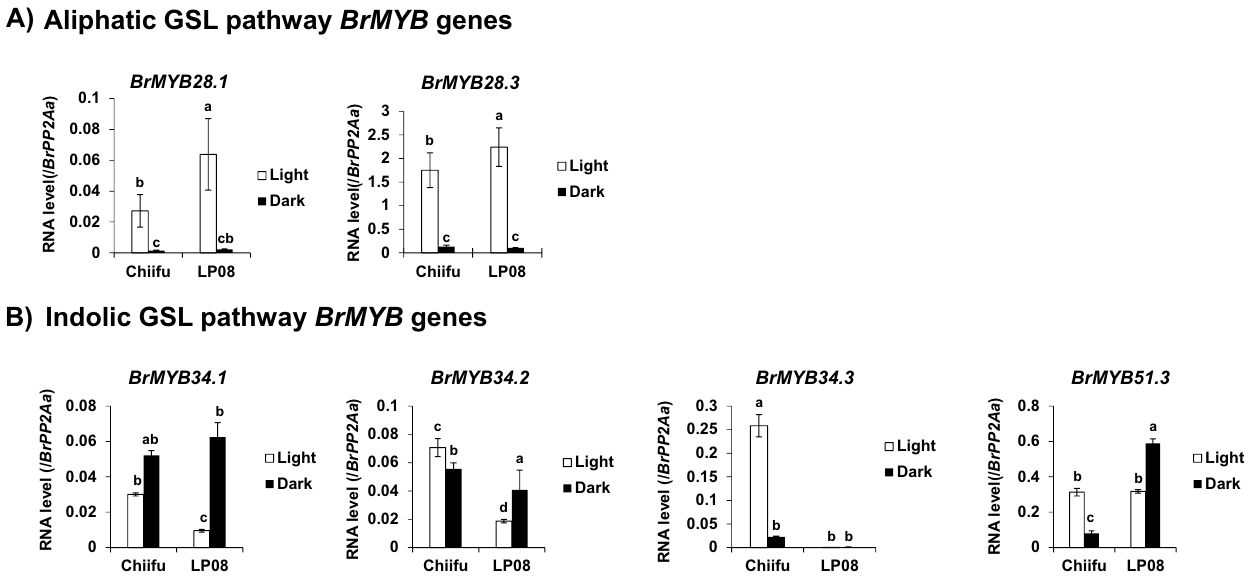

Supplement: Supplementary Figure 1 — UHPLC chromatogram of GSLs extracted from seedlings of ‘LP08’ (upper) and ‘Chiifu’ (bottom). 1, progoitrin (PGT); 2, glucoraphanin (GRA); 3, glucoalyssin (GAS); 4, gluconapoleiferin (GNA); 5, gluconapin (GNP); 6, 4-hydroxyglucobrassicin (4-HGB); 7, glucobrassicanapin (GBN); 8, glucoerucin (GER); 9, glucobrassicin (GBC); 10, 4-methoxyglucobrassicin (4-MTGB); 11, neoglucobrassicin (NGB); 12, gluconasturtiin (GNT). [file DataSheet_1.zip › Supplementary figure S/Supp Fig. S4AB.tif]

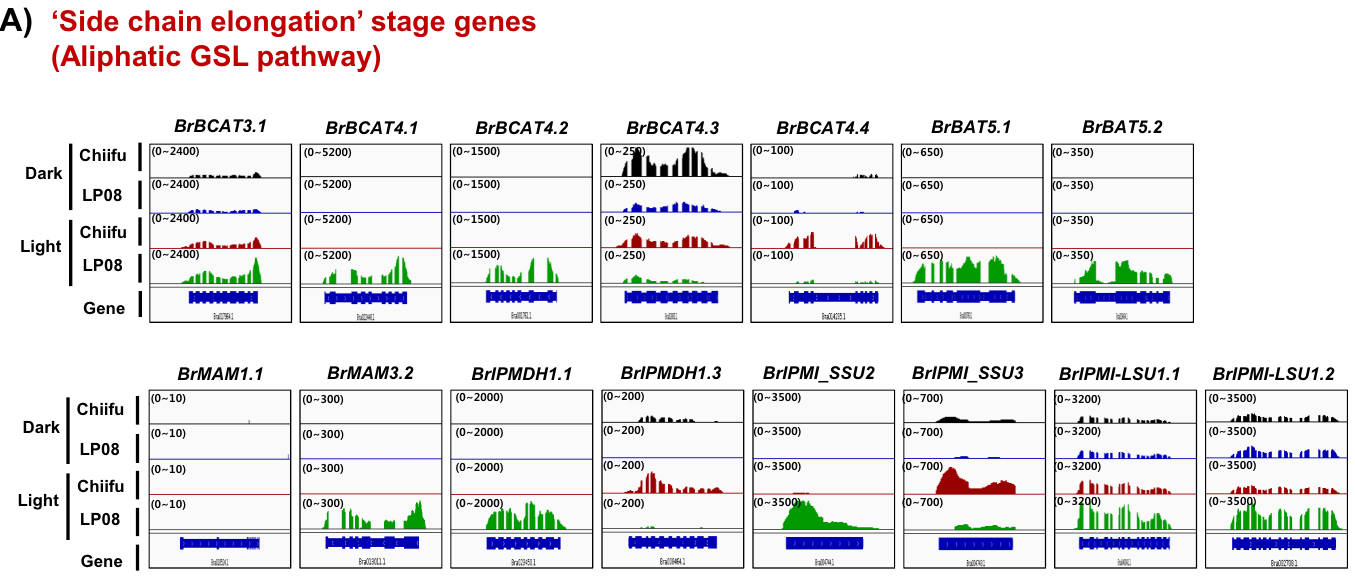

Supplement: Supplementary Figure 1 — UHPLC chromatogram of GSLs extracted from seedlings of ‘LP08’ (upper) and ‘Chiifu’ (bottom). 1, progoitrin (PGT); 2, glucoraphanin (GRA); 3, glucoalyssin (GAS); 4, gluconapoleiferin (GNA); 5, gluconapin (GNP); 6, 4-hydroxyglucobrassicin (4-HGB); 7, glucobrassicanapin (GBN); 8, glucoerucin (GER); 9, glucobrassicin (GBC); 10, 4-methoxyglucobrassicin (4-MTGB); 11, neoglucobrassicin (NGB); 12, gluconasturtiin (GNT). [file DataSheet_1.zip › Supplementary figure S/Supp Fig. S5A.tif]

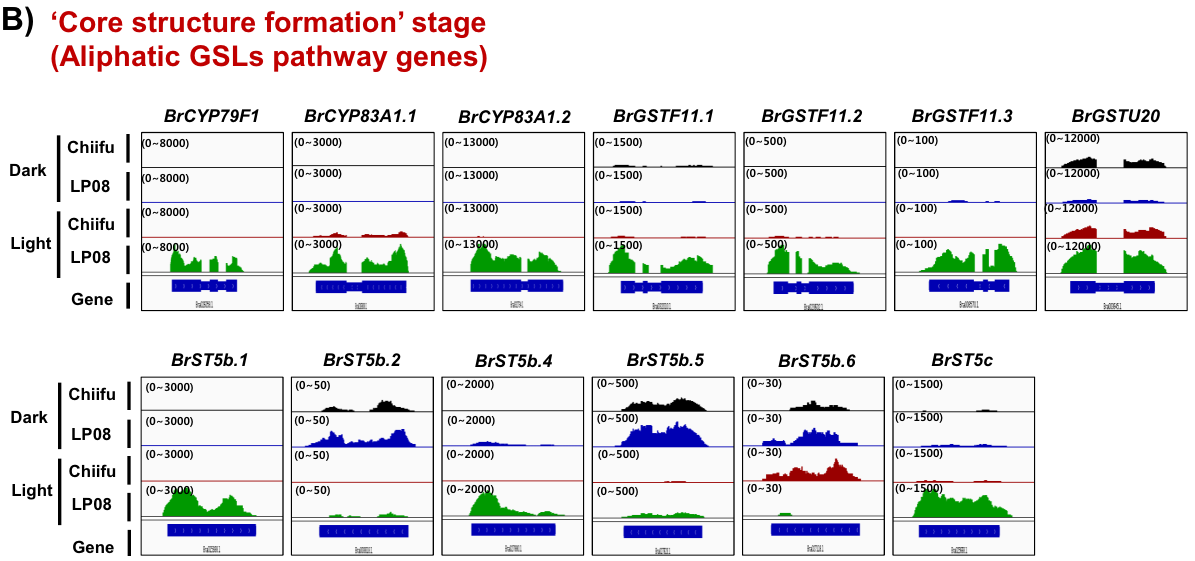

Supplement: Supplementary Figure 1 — UHPLC chromatogram of GSLs extracted from seedlings of ‘LP08’ (upper) and ‘Chiifu’ (bottom). 1, progoitrin (PGT); 2, glucoraphanin (GRA); 3, glucoalyssin (GAS); 4, gluconapoleiferin (GNA); 5, gluconapin (GNP); 6, 4-hydroxyglucobrassicin (4-HGB); 7, glucobrassicanapin (GBN); 8, glucoerucin (GER); 9, glucobrassicin (GBC); 10, 4-methoxyglucobrassicin (4-MTGB); 11, neoglucobrassicin (NGB); 12, gluconasturtiin (GNT). [file DataSheet_1.zip › Supplementary figure S/Supp Fig. S5B.tif]

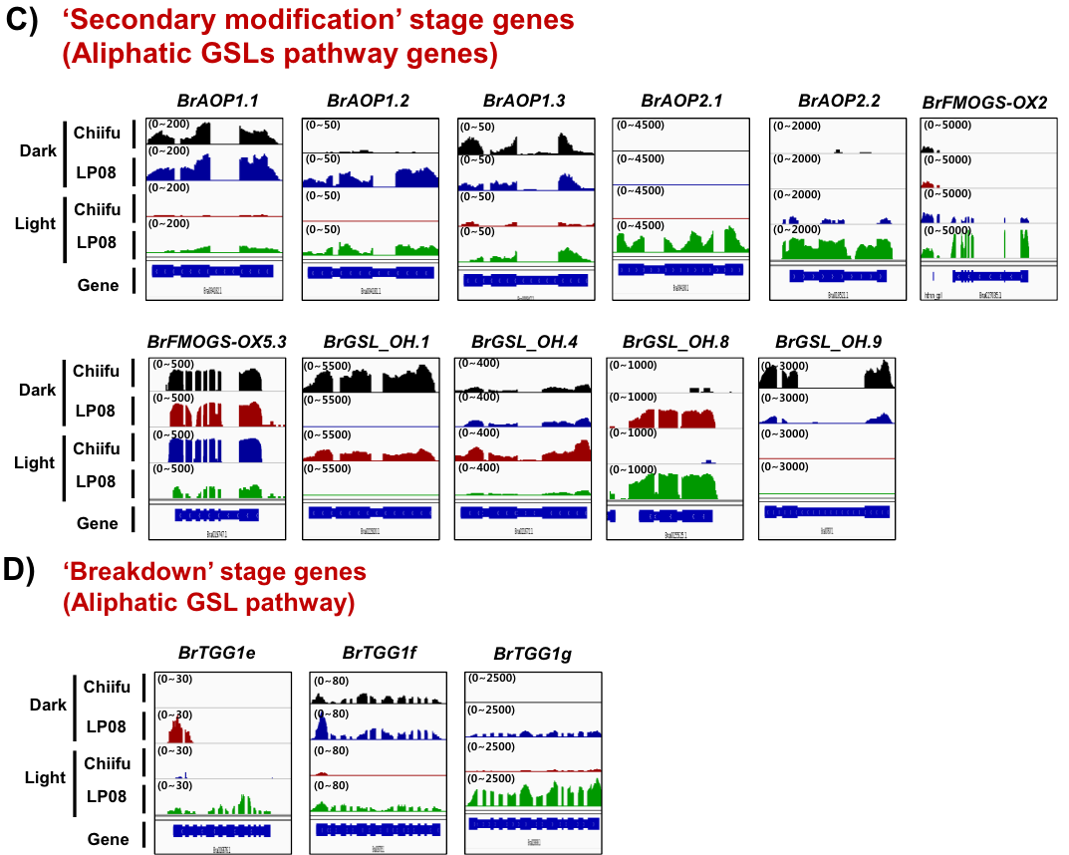

Supplement: Supplementary Figure 1 — UHPLC chromatogram of GSLs extracted from seedlings of ‘LP08’ (upper) and ‘Chiifu’ (bottom). 1, progoitrin (PGT); 2, glucoraphanin (GRA); 3, glucoalyssin (GAS); 4, gluconapoleiferin (GNA); 5, gluconapin (GNP); 6, 4-hydroxyglucobrassicin (4-HGB); 7, glucobrassicanapin (GBN); 8, glucoerucin (GER); 9, glucobrassicin (GBC); 10, 4-methoxyglucobrassicin (4-MTGB); 11, neoglucobrassicin (NGB); 12, gluconasturtiin (GNT). [file DataSheet_1.zip › Supplementary figure S/Supp Fig. S5CD.tif]

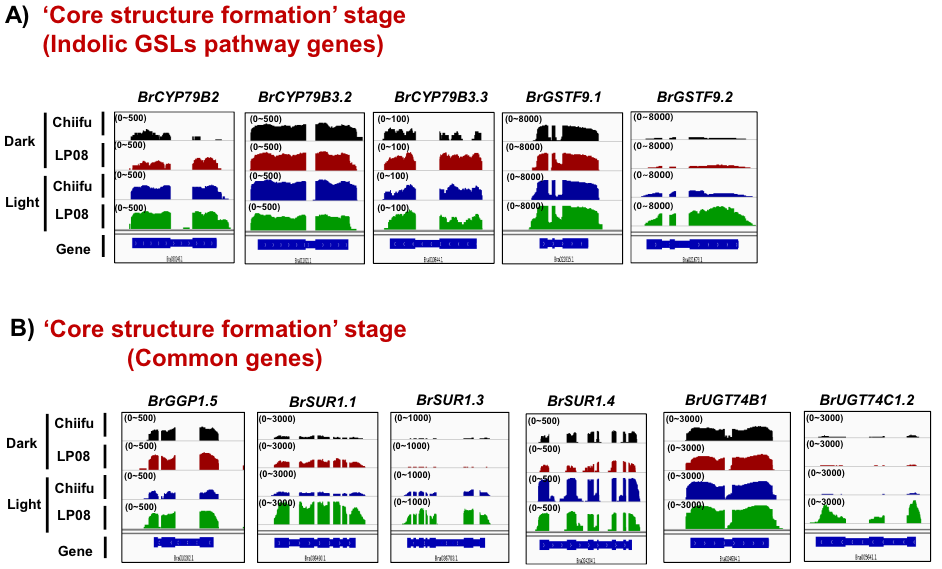

Supplement: Supplementary Figure 1 — UHPLC chromatogram of GSLs extracted from seedlings of ‘LP08’ (upper) and ‘Chiifu’ (bottom). 1, progoitrin (PGT); 2, glucoraphanin (GRA); 3, glucoalyssin (GAS); 4, gluconapoleiferin (GNA); 5, gluconapin (GNP); 6, 4-hydroxyglucobrassicin (4-HGB); 7, glucobrassicanapin (GBN); 8, glucoerucin (GER); 9, glucobrassicin (GBC); 10, 4-methoxyglucobrassicin (4-MTGB); 11, neoglucobrassicin (NGB); 12, gluconasturtiin (GNT). [file DataSheet_1.zip › Supplementary figure S/Supp Fig. S6AB.tif]

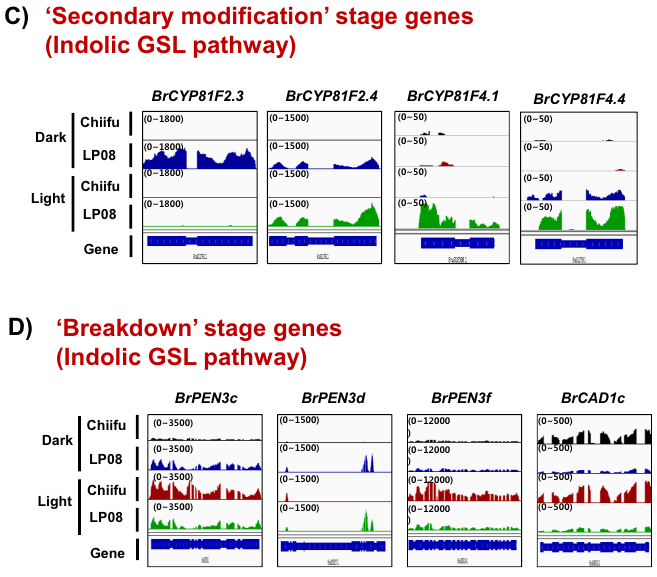

Supplement: Supplementary Figure 1 — UHPLC chromatogram of GSLs extracted from seedlings of ‘LP08’ (upper) and ‘Chiifu’ (bottom). 1, progoitrin (PGT); 2, glucoraphanin (GRA); 3, glucoalyssin (GAS); 4, gluconapoleiferin (GNA); 5, gluconapin (GNP); 6, 4-hydroxyglucobrassicin (4-HGB); 7, glucobrassicanapin (GBN); 8, glucoerucin (GER); 9, glucobrassicin (GBC); 10, 4-methoxyglucobrassicin (4-MTGB); 11, neoglucobrassicin (NGB); 12, gluconasturtiin (GNT). [file DataSheet_1.zip › Supplementary figure S/Supp Fig. S6CD.tif]

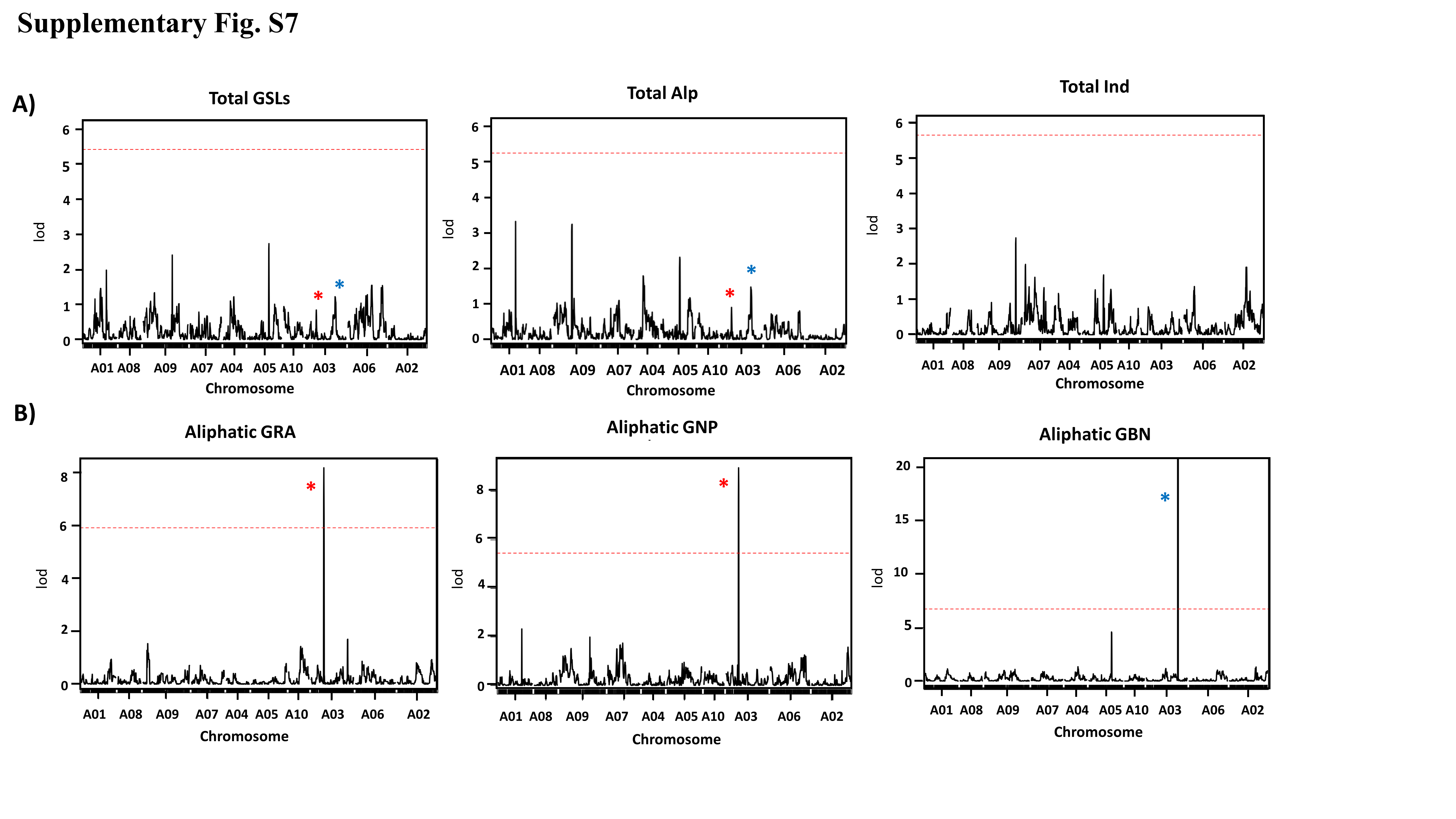

Supplement: Supplementary Figure 1 — UHPLC chromatogram of GSLs extracted from seedlings of ‘LP08’ (upper) and ‘Chiifu’ (bottom). 1, progoitrin (PGT); 2, glucoraphanin (GRA); 3, glucoalyssin (GAS); 4, gluconapoleiferin (GNA); 5, gluconapin (GNP); 6, 4-hydroxyglucobrassicin (4-HGB); 7, glucobrassicanapin (GBN); 8, glucoerucin (GER); 9, glucobrassicin (GBC); 10, 4-methoxyglucobrassicin (4-MTGB); 11, neoglucobrassicin (NGB); 12, gluconasturtiin (GNT). [file DataSheet_1.zip › Supplementary figure S/Supp Fig. S7AB.tif]

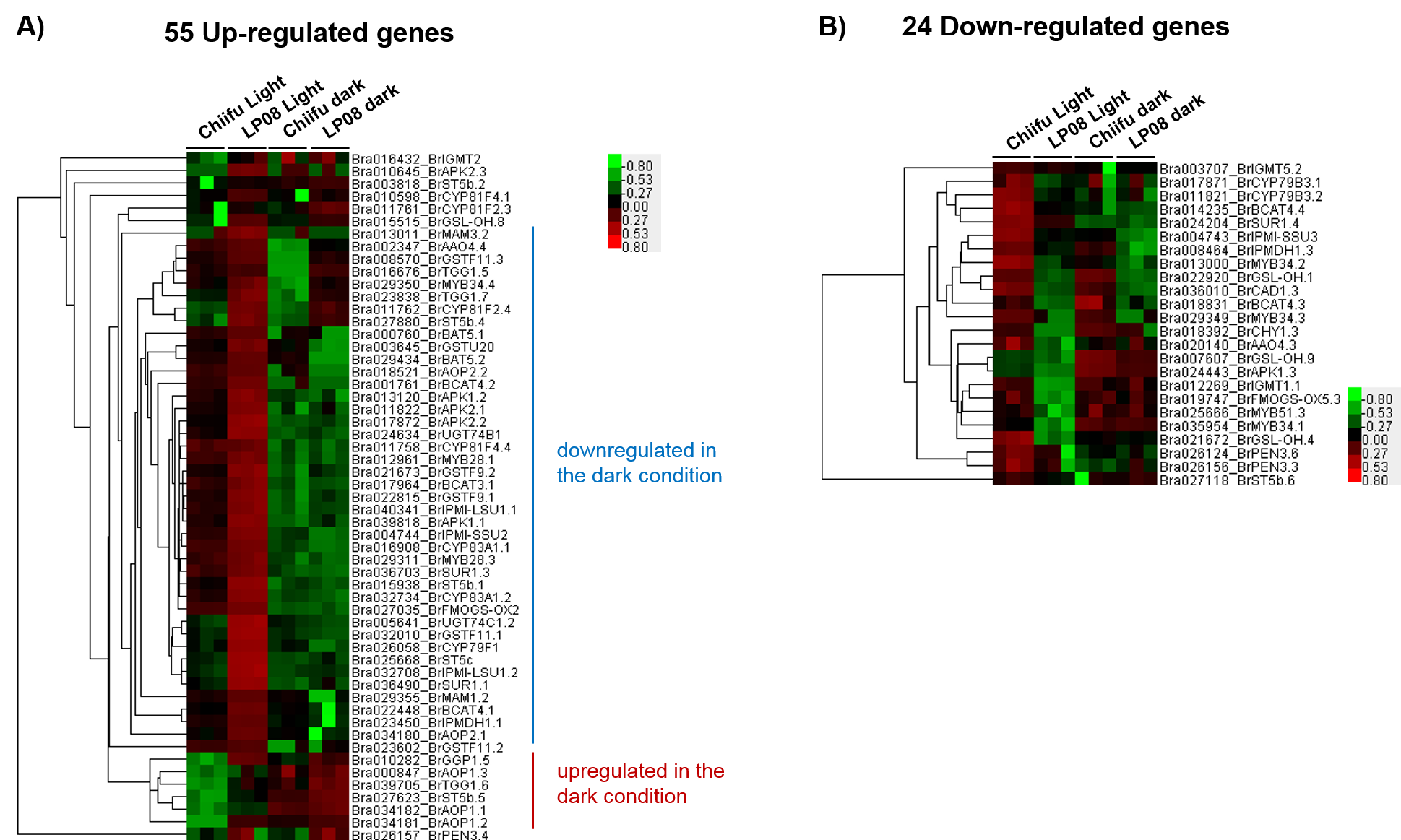

Supplement: Supplementary Figure 1 — UHPLC chromatogram of GSLs extracted from seedlings of ‘LP08’ (upper) and ‘Chiifu’ (bottom). 1, progoitrin (PGT); 2, glucoraphanin (GRA); 3, glucoalyssin (GAS); 4, gluconapoleiferin (GNA); 5, gluconapin (GNP); 6, 4-hydroxyglucobrassicin (4-HGB); 7, glucobrassicanapin (GBN); 8, glucoerucin (GER); 9, glucobrassicin (GBC); 10, 4-methoxyglucobrassicin (4-MTGB); 11, neoglucobrassicin (NGB); 12, gluconasturtiin (GNT). [file DataSheet_1.zip › Supplementary figure S/Supp Fig. S8AB.tif]
